# Risk Factors of Severe COVID-19: A Review of Host, Viral and Environmental Factors

**DOI:** 10.3390/v15010175

**Published:** 2023-01-07

**Authors:** Levente Zsichla, Viktor Müller

**Affiliations:** 1 Institute of Biology, Eötvös Loránd University, 1117 Budapest, Hungary; 2 National Laboratory for Health Security, Eötvös Loránd University, 1117 Budapest, Hungary

**Keywords:** COVID-19, disease severity, risk factors, age, sex, comorbidities, coinfections, host genetics, viral variants, socioeconomic status

## Abstract

The clinical course and outcome of COVID-19 are highly variable, ranging from asymptomatic infections to severe disease and death. Understanding the risk factors of severe COVID-19 is relevant both in the clinical setting and at the epidemiological level. Here, we provide an overview of host, viral and environmental factors that have been shown or (in some cases) hypothesized to be associated with severe clinical outcomes. The factors considered in detail include the age and frailty, genetic polymorphisms, biological sex (and pregnancy), co- and superinfections, non-communicable comorbidities, immunological history, microbiota, and lifestyle of the patient; viral genetic variation and infecting dose; socioeconomic factors; and air pollution. For each category, we compile (sometimes conflicting) evidence for the association of the factor with COVID-19 outcomes (including the strength of the effect) and outline possible action mechanisms. We also discuss the complex interactions between the various risk factors.

## 1. Introduction

Coronavirus disease 2019 (COVID-19) has affected all human populations worldwide, but its toll in mortality and morbidity has been distributed very unevenly across geographical regions, across age groups, and along the spectra of other host, viral and environmental factors. Characterizing the factors that are associated with the outcome of infections with severe acute respiratory syndrome coronavirus 2 (SARS-CoV-2) has relevance at multiple levels. First, an understanding of these factors is required for the assessment of the risk of severe disease in individual patients, which may guide therapeutic decisions in patient care. Antiviral treatments against SARS-CoV-2 are most effective when administered early, before the onset of severe symptoms [1,2]; however, the cost, availability and side-effects of the treatments preclude broad prophylactic application. Under these conditions, effective treatment protocols require reliable prognosis in the early stages of infection, which can be aided by considering known risk factors.

Second, well-characterized risk factors, combined with the prevalence and distribution of these factors in a population can be used to forecast potential mortality and morbidity at the population level, which can inform policy decisions, and guide an optimized public health response.

Third, while a statistical association (between a potential risk factor and COVID-19 outcome) does not necessarily imply causality, identified risk factors may provide clues for causative mechanisms of pathogenesis. Elucidating these mechanisms can guide the development of new therapeutic options, as well as effective non-pharmaceutical interventions against COVID-19.

We provide a structured overview of host, viral and environmental factors that have been shown to be associated with severe clinical outcomes. These associations are typically quantified in terms of risk, odds, or hazard ratios—we provide a brief explanation of these terms in Appendix A.

## 2. Host Factors

### 2.1. Age

Age is among the strongest risk factors of COVID-19 mortality. This effect was first reported in early 2020 [3,4], and has since been confirmed by several meta-analyses [5,6]. The risk of death in particular is best reflected by the infection fatality ratio (IFR, probability of death upon infection) of SARS-CoV-2, which has been reliably estimated in relation to age for the first waves of the pandemic by combining information on age-specific mortality and seroprevalence data [7,8,9]. These results indicate that in adults, the IFR increases exponentially with age, doubling the risk of death with approximately every 6–7 years of age, and (for the first large wave of the pandemic) exceeding 1% between 65 and 75 years [9]. In addition to increased mortality, older patients typically experience more severe symptoms [5,10,11] and require hospitalization [12,13], intensive care [5,11,12] and mechanical ventilation (MV) [11,12] more often.

Age-related changes in the human body can affect COVID-19 pathogenesis in a multitude of ways. Aging of the lungs involves increased cellular senescence, epigenetic dysregulation, oxidative stress, mitochondrial dysfunction, inflamm-aging and immunosenescence associated with NK cell cytotoxicity and immune surveillance [14]. Several immunological changes connected to aging may also exacerbate COVID-19 pathogenesis such as altered IFN-γ signaling; neutrophilic infiltration; decreased CD4+ or CD8+ T cell, and naïve B cell levels; alveolar macrophage activation; and elevated release of pro-inflammatory cytokines [14]. Shorter telomeres have also been linked to COVID-19 severity [15]. Moreover, an analysis using single-cell transcriptomics data from multiple cell types identified a handful of genes that are progressively upregulated with age, are dysregulated by SARS-CoV-2 infection, and have important roles both in the aging of the lung and in the pathogenesis of COVID-19 [16]. The genes identified are involved in altered immune cell recruiting, impaired mitochondrial functions, and increased neutrophil attraction (neutrophil extracellular trap (NET) formation).

Age-related changes in the expression of the SARS-CoV-2 cellular entry receptor ACE2 have also been suggested to contribute to the severity of COVID-19. Although pre-COVID-19 analyses reported no significant difference in ACE2 levels according to age [17,18], ACE2 expression in the lungs of COVID-19-infected patients has been demonstrated to correlate with age [16,19], and disease severity also showed an association with ACE2 levels in the respiratory system [20,21]. Furthermore, chronic comorbidities also positively correlated with ACE2 levels in multiple analyses [22,23], which might explain the higher severity of disease in these risk groups. In turn, a higher expression of ACE2 may also counteract oxidative stress and inflammation through the role of the receptor in the renin–angiotensin system (RAS) [24,25], although the significance of this anti-inflammatory mechanism on COVID-19 severity is not well supported by clinical data. The increased expression of TMPRSS2 with age (the serin protease responsible for priming the SARS-CoV-2 spike protein of pre-omicron variants) [26,27] has also been linked to the greater susceptibility of adults to severe COVID-19 [28,29].

Decreased apoptotic sensitivity of aged lung tissue after viral infection has also been implicated in the age dependence of COVID-19 [19]. Early apoptosis mitigates SARS-CoV-2 production [30,31] and, in the case of other infections, it has been shown to decrease both disease severity and mortality [32].

Inflamm-aging is a progressive immunophysiological process associated with increased levels of basal inflammatory mediators (such as IL-6, IL-1β, TNF-α and CRP [33]) driven by the stimulation of the NF-κB signaling pathway and mediated by immune and senescent cells [34]. Severe COVID-19 and advanced age are both correlated with biomarkers of systemic inflammation [35], such as the neutrophil/leukocyte ratio (NLR) [36], weaker type-I IFN responses [37], NLRP3 inflammasome activation [38], and IL-6, IL-12 and IL-1β secretion [39]. Inflamm-aging is a part of a diverse set of mechanisms responsible for the progressive development of hyporesponsiveness and dysregulation of immunity during aging [34,40], called immunosenescence, which is believed to be a major driving force of COVID-19 pathogenesis [41]. Dysregulated immune processes correlated with severe COVID-19 include deficient early type-I IFN production, dysregulated inflammation by neutrophils and monocytes and diminished T cell responses [33,42].

Chronological age is strongly correlated with the molecular and physiological mechanisms of aging but does not reflect individual variation in the rate of these processes. To overcome this problem, several markers of ‘biological age’ have been proposed (telomere length, transcriptomic and metabolic signals, composite markers, etc.), second-generation epigenetic clocks (e.g., PhenoAge and GrimAge) being the most reliable estimators of all-cause mortality and aging-related physiological changes [43,44]. According to recent evidence, telomere length shortening [45,46] and the acceleration of epigenetic age (independent of chronological age) [45,47] are both associated with adverse outcomes during COVID-19. Epigenetic markers of disease severity are concentrated near promoter regions (including the promoters of certain aging-related genes [48]) and include the hypermethylation of IFN-related and the hypomethylation of inflammatory genes [49,50]. In turn, COVID-19 might influence the markers included in biological age estimators and possibly, the process of aging as well [51,52].

Finally, although other respiratory viral infections (e.g., respiratory syncytial virus (RSV) and influenza) affect children more severely compared to adults [53,54], most children are protected against severe COVID-19. Potential contributing factors include the low prevalence of chronic comorbidities [55], strong and rapid innate immune responses [56,57] and a more naïve character of immunity (higher ratio of naïve T cells, lower levels of cytotoxic T cells and NK cells and less T cell exhaustion [58]) compared to adults and especially to the elderly [59]. The observed robust innate immunity in children might be the consequence of differences in IFN responses resulting from the dominance of Orf1ab-specific CD4+ T cells [60], which act against non-structural proteins diminishing IFN signalization [61] or higher trained immunity resulting from frequent viral infections during childhood [62]. We note that all these factors may also affect the course of other respiratory viral infections, and the lower severity typically observed in adults with endemic respiratory viruses may be a consequence of pathogen-specific adaptive immunity acquired in childhood, which was also evidently absent in adults against SARS-CoV-2 during the first waves of the pandemic.

We conclude that the strong impact of age on the outcome of COVID-19 in adults may be a combined effect of multiple factors associated with aging.

### 2.2. Human Genetic Variation

Hundreds of thousands of SARS-CoV-2-infected cases have been screened for genetic information using whole-genome or whole-exome sequencing, or genotyping microarrays. These analyses have identified dozens of genes/loci that appear to be correlated with COVID-19 severity; in Table 1, we list the most plausible candidates that have either been confirmed by independent analyses and/or that have been regularly listed in reviews based on both bioinformatic and empirical studies.

The variants identified by high-throughput genetic screening might affect COVID-19 by interfering with viral entry, by modulating antiviral immunity, or by modulating the renin–angiotensin system responsible for the regulation of blood pressure that SARS-CoV-2 interferes with through its binding to ACE2; for some other variants, the mechanistic link is less clear. The effect sizes (odds ratios, OR) for individual variants ranged between OR = 0.819 (0.781–0.858) and OR = 1.885 (1.748–2.032) for critical illness, and between OR = 0.861 (0.834–0.889) and OR = 1.649 (1.562–1.741) for hospitalization in the largest GWAS study to date [83]. In another study, the overall effect of human genetic variation was assessed by estimating a polygenic risk score, and a high risk score was associated with a higher risk of severe COVID-19 compared with individuals with low genetic risk score (OR = 1.50 (1.18–1.92)) [84].

While genome-wide screening is able to identify genetic effects in an unbiased, systematic way, it has limited power to detect the effect of rare genetic variants [85]. Several rare genetic disorders have been implicated in severe COVID-19 by targeted analyses of a heterogeneous group of genetic variants called inborn errors of immunity (also called primary immunodeficiencies) [86]. The strongest candidates are loss-of-function variants of the X-chromosomal TLR7 gene (see also Table 1), which are associated with impaired IFN I and III responses, and are enriched in males under 60 years with critical COVID-19 outcomes [76,87,88,89]. A large study found that antiviral interferon responses might be compromised in at least 3.5% of patients with severe COVID-19 pneumonia via other components of the signaling pathway, such as TLR3, IRF7 or IFNAR1 [90], but independent analyses failed to reproduce the association of these rare alleles with severe disease [91,92]. While the impact of individual variants is hard to estimate due to the low sample sizes, a systematic review of primary immunodeficiencies estimated an overall hospitalization rate of 49%, and a case fatality rate of 9% for all cases combined [93].

Finally, although the role of host genetics is unknown in this case, the role of interferon signaling and the renin–angiotensin system in COVID-19 is further supported by the observation that IFN-I-neutralizing [94] and AngII autoantibodies [95] can be detected in a significant portion of deceased and hospitalized COVID-19 patients, respectively.

Most of the identified rare variants are associated with an increased probability of adverse clinical outcomes in COVID-19. While there is no known genetic variant that would offer complete resistance against SARS-CoV-2 infection [96,97], a small number of candidate variants might decrease the severity of disease (Table 1). An exonic mutation in TMPRSS2 [64,98], an indel in ACE1 [64], a mucus-oversecreting MUC5B variant [67,69,99], some HLA class 1 alleles [100,101,102] and blood group O (possibly by lowering the risk of cardiovascular complications) [103] may have a protective effect against severe COVID-19.

Several studies have addressed the impact of ancestry, which defines the large-scale patterns of human genetic variation, on the risk of severe COVID-19 [104,105,106]. However, while non-European ancestry has been repeatedly linked to a higher risk of severe outcomes [107], it seems unlikely that genetic differences account for the observed effect [67,68]. Instead, ancestry is often correlated with socioeconomic factors that affect COVID-19 outcomes (see Section 4.1. *Socioeconomic factors*), and the statistical effect of ancestry appears to be explained by correlated socioeconomic variables [106,108,109]. A rare known example related to ancestry is presented by some genetic components that have entered the human genome through Neanderthal introgression, such as the 3p21.31 locus (LTZFL1/SLC6A20) that increases [110], and the OAS cluster that decreases [111] the risk of severe COVID-19. Accordingly, both variants are more common in European and South Asian compared to African and East Asian populations.

We conclude that a few human genetic polymorphisms might have a relatively strong negative impact on the outcome of COVID-19, but most individual variants, and ancestry/ethnicity, appear to have no or only small effect.

### 2.3. Sex and Pregnancy

Certain respiratory viral infections disproportionately affect the two sexes. Males typically experience more frequent lower respiratory tract infections [112] and, in the case of influenza A and B viruses, RSV, SARS-CoV, and other sources of community-acquired pneumonia, more severe disease compared to females [113,114]. Similarly, with SARS-CoV-2 infection, males are hospitalized [8,115], are admitted to intensive care unit (ICU) [8,116] and die [117,118] more often, although infection rates appear to be similar to those of women [119]. Sex-disaggregated estimates of the infection fatality ratio show consistently higher fatality rates among males in every age category [8,9]. We summarize some of the largest studies and meta-analyses demonstrating the association of COVID-19 outcomes with biological sex in Table 2.

The underlying causes (which might apply for respiratory infections in general) might include differences in the prevalence of comorbidities [125], lifestyle choices [126], immunogenetic [127] and immunoendocrine [128] factors.

Sex hormones alter the expression of certain genes (through interaction with hormone response elements) related to physiological and immunological functions [129]. Hormonal differences between males and females may thus be partly responsible for sex-specific differences in the responses to respiratory viral infections and, particularly, COVID-19 [130].

Androgens modulate immune and inflammatory processes, typically in an immunosuppressive and anti-inflammatory manner [131]. In COVID-19 patients, this immune-modulating role may even be beneficially connected to the pathogenesis of disease, indicated by the observation that low levels of the major androgen, testosterone (which is a natural consequence of aging in males [132]) are associated with pro-inflammatory states and unfavorable disease outcomes, such as severe symptoms, acute respiratory distress syndrome (ARDS) and ICU admission, even when controlling for age [133,134]. Lastly, a genetic polymorphism, the length of the N-terminal polyQ tract in the androgen receptor is correlated with COVID-19 severity, with shorter alleles implying higher vulnerability against adverse outcomes [135]. Possible mechanisms explaining the negative impact of low testosterone are enhanced replication as a result of the SARS-CoV-2-induced impairment of intracellular Ca^2+^-regulation [136] and increased pro-inflammatory activity of innate immune cells [137]. In turn, androgens might also facilitate viral entry by the induction of TMPRSS2 expression [138].

Postmenopausal women also showed increased severity of COVID-19 in a small study [139], which may be partly attributed to low estrogen production [140], which is associated with higher risk of COVID-19 hospitalization and increased systemic inflammation [141]. Although age-related changes in sexual functions are difficult to disentangle from other effects of aging, several hypotheses have been proposed for the possible role of sex-linked factors. Estrogen hormones influence the expression of SARS-CoV-2 entry receptors, increasing ACE2 [142] and decreasing TMPRSS2 [143] levels; they induce innate [144] and adaptive [145] immune cells, promote anti-inflammatory functions [144], influence insulin secretion [146] and protection against cardiovascular diseases (CVDs) [147].

In addition to the influence of sex hormones, some X-linked genes may also contribute to sex-specific differences in COVID-19 immunity and outcome. The random inactivation of one of two X chromosomes in females creates variation in the expression of both alleles (‘X mosaicism’). Furthermore, this inactivation might be incomplete in some cases, which increases gene dosage in females. The gene of ACE2 is located on the X chromosome and displays X mosaicism. Although ACE2 expression is similar in respiratory tissues between the two sexes [148,149], age-related declines of its expression might be more pronounced in men [150]. Remarkably, while the respiratory expression of TMPRSS2 is also similar between the sexes [148], cells co-expressing both ACE2 and TMPRSS2 are 3-fold more abundant in males [151]. However, the role of these sex-specific differences in SARS-CoV-2 entry receptor expression on the observed clinical outcomes is unclear. The immune receptor TLR7 shows incomplete X-inactivation with higher expression and downstream signaling in females and reduced functions in males [152]. The Y chromosome is also involved in immune functions, altering gene expression in CD4+ T cells and macrophages [153]. Furthermore, loss of Y chromosome in immune cells is associated with impaired lymphoid functions [154,155].

Adaptive immune responses in women are also stronger against SARS-CoV-2 [156], indicated by greater IFN signalization [157], more robust T cell activation [158,159,160], and effective humoral immunity [161,162]. Moreover, autoantibody production against IFNs [163,164], and inborn errors of immunity such as impaired IFN signalization [76] (see Section 2.2. *Human Genetic Variation*) are also more often found in male patients. These factors might explain the observed lower concentration of pro-inflammatory (NLR, CRP, IL-6, and TNF-α) [161,165] and cellular damage markers (ALT and AST) [158] in women compared with men.

Although female patients are somewhat more protected against unfavorable outcomes of COVID-19, they are more severely affected by its long-term complications [166] and by lifestyle changes in the COVID-19 era [167].

Finally, pregnancy is a risk factor of severe COVID-19 that only affects women. Pregnant women show increased susceptibility to several viruses such as influenza A [168], SARS-CoV and MERS-CoV [169]. Similarly, even though the overall risk of unfavorable outcomes is rare [170,171,172], the risk of severe outcomes such as pneumonia [173], ICU admission [173,174], MV [174] and mortality [175,176] seems to be higher in pregnant, compared with non-pregnant women. In addition to the effect of pregnancy on the severity of COVID-19, SARS-CoV-2 infection also seems to influence the risk of complications in pregnancy. Compared with pregnant SARS-CoV-2-negative women, pregnant infected women more often develop hypertensive disorders (preeclampsia, eclampsia) [177,178], are admitted to ICU [174,179,180], receive MV [181] or die [174]. Adverse outcomes also affect the development of the fetus. Before or during delivery, fetuses of SARS-CoV-2-positive women are more likely to experience hyperbilirubinemia [182], intrapartum fetal distress [183], cesarean delivery [180,184,185], fetal growth restriction (resulting in low birthweight) [184], preterm birth [180,184,186,187] and stillbirth [177,186,187]. SARS-CoV-2 infection early in gestation seems to produce more severe consequences; however, most SARS-CoV-2-positive women are in the second or third trimester at the time of diagnosis [187]. Newborns of SARS-CoV-2-infected mothers have an increased risk of ARDS [182], ICU admission [174,180,182] and neonatal death [176].

Pregnancy is accompanied by complex endocrine, physiological, and immunological changes which might affect COVID-19 pathogenesis. While increased severity of COVID-19 in pregnant women is not properly understood, SARS-CoV-2 infection may damage the fetus in direct and indirect ways. The co-expression of ACE2 and TMPRSS2 on placental cells is rare [188], but several alternative SARS-CoV-2 entry receptors (CTSL, CTSB, and BSG/CD147) are abundant on multiple placental cell types [189]. Direct infection of the placenta is possible ex vivo [190], but seems to be rare in vivo [191]. However, the rare cases of viral invasion can involve extensive areas of the placenta [192]. Similarly, intrauterine vertical transmission of SARS-CoV-2 has been documented [193], but seems to be rare [194,195], possibly due to the relatively low viraemia in COVID-19 patients [196]. Inflammatory markers have been found in umbilical cord blood [197], indicating that indirect damage to the placenta and to the fetus by pro-inflammatory cytokines might also contribute to neonatal complications [198]. Whether directly or indirectly, damage to the placenta might induce a severe hypoxic state responsible for the adverse effects of COVID-19 [199]. Inflammation due to SARS-CoV-2 infection might also contribute to cytokine-driven neonatal respiratory distress in the fetus [200].

To conclude, compelling evidence supports that both male biological sex and pregnancy are strong risk factors of severe COVID-19, with many possible action mechanisms, but no compelling evidence on the relative importance of each.

### 2.4. Comorbidities

#### 2.4.1. Non-Communicable Diseases (NCDs)

Chronic non-communicable diseases often involve the deterioration of one or multiple physiological functions, which might modulate the course of COVID-19. Indeed, many common NCDs, including cardiovascular, chronic respiratory or metabolic diseases, and various types of cancer, have been consistently associated with an increased risk of poor clinical outcomes in SARS-CoV-2-infected individuals (Table 3), while for some other comorbid conditions the evidence is less clear (Table 4).

Chronic obstructive pulmonary disease (COPD) was the third leading cause of death worldwide in 2019 [210], and this common condition has been associated with an increased risk of hospitalization, ICU admission and in-hospital death in COVID-19 [203,211]. The mechanisms potentially involved in the increased severity of COVID-19 in COPD patients are multifold. COPD is characterized by abnormal lung structure, impaired tissue repair, and severe loss of respiratory function [212,213]. Dysfunctional innate and adaptive immune responses [214,215,216], increased chronic inflammation [212,216] and upregulated ACE2 expression [213,216,217,218] have also been hypothesized to contribute to the risk of severe COVID-19. Exacerbation of lung pathology by other respiratory infections is common in COPD patients [219,220,221,222,223,224], suggesting shared mechanisms. It is also important to note that COPD frequently coexists with other NCDs [225,226,227] and its prevalence increases with age [228]. However, the studies controlling for age [3,7] or comorbidities [3] still found a significant independent effect of COPD on COVID-19 outcomes.

Interstitial lung disease (ILD) is a heterogeneous group of chronic conditions involving endothelial (alveolar) injury and fibrosis resulting in impaired gas exchange and limited pulmonary reserve [229,230], and has been associated with an increased risk of severe disease [231], ICU admission [232] or death [232,233,234] in COVID-19. In addition to the direct effect of impaired respiratory functions and resilience, the use of immunosuppressive medications in ILD has also been hypothesized to contribute to the observed increased likelihood of COVID-19 mortality [232].

Contrary to expectations, asthma has failed to show a clear effect on COVID-19 outcomes [235]. Both COPD and asthma are chronic lung diseases involving shortness of breath, chest tightness, wheezing and cough, but the causes and mechanisms of the two conditions are largely different. As opposed to COPD, in allergic (atopic) asthma ACE2 levels are decreased both in upper and lower airways [216,236,237], inflammation is characterized by a type 2 [238] instead of a type 1 response, and T cell levels are not decreased [26,52]. A component of type 2 inflammation, eosinophilia has been shown to be independently associated with decreased severity of COVID-19 [239]. In turn, other respiratory viral infections (influenza, rhinovirus, respiratory syncytial virus) have been associated with the exacerbation of asthma [240,241,242], and impaired IFN signalization has been observed in cells obtained from asthmatic patients upon viral infection [243,244,245]. Asthma is also a risk factor for hospitalization after influenza infection [246]. Fortunately, an aggravating effect has not been detected in SARS-CoV-2-infected patients with atopic asthma. However, non-atopic asthma has been associated with exacerbated symptoms [247,248]. It is characterized by type 1 inflammation (without eosinophilia) and is associated with age and other NCDs [247].

The associated conditions of obesity, diabetes, hypertension and cardiovascular disease (CVD) have all been consistently shown to considerably increase the risk of severe COVID-19 outcomes [201,202]. Given the high worldwide prevalence of these conditions (e.g., 13% of all adults globally have been estimated to be obese in 2020 [249]), they may have had the greatest contribution to COVID-19 mortality among all comorbidities, and may have only been exceeded by the effect of old age overall. In the most likely scenario, obesity, diabetes, hypertension and CVD are all part of the same interconnected pathophysiological pathway [250,251]. The interrelated nature of these chronic diseases is supported by their similarly high prevalence and co-occurrence in MERS and SARS patients [251,252,253]. An individual’s genetic background, existing insulin resistance, dyslipidemia and obesity are main risk factors of diabetes and hypertension, which result in hyperglycemia (which is associated with COVID-19 severity independently from diabetes [254,255,256]), dysregulation of the RAS, heightened immune activation, oxidative stress, and chronic inflammation (IL-1β, IL-6, and TNF-α) [250]. These complex changes give rise to chronic CVD. During COVID-19 pulmonary distress puts an increasing burden on the previously weakened cardiovascular system with a damaged pulmonary endothelial barrier, fluid extravasation, hypoxia, heightened inflammation (possibly through the decreased airway ACE2 levels in CVD patients [216,236,237]) and hypercoagulability, which culminate in possible acute consequences such as myocardial injury, infarction, heart failure, thrombosis or arrythmias [251,257,258,259]. Obesity is further associated with an increased risk of obstructive sleep apnea, asthma, and COPD [260,261,262,263] and adipose tissue seems to be a direct target of SARS-CoV-2 replication further exacerbating hyperglycemia and hyperinsulinemia [264].

Chronic kidney disease (CKD) has been associated with the severity of pneumonia [265], and it is among the strongest risk factors for hospitalization [266] and in-hospital death [201,202,206] with COVID-19. Similarly to cells of the heart and vascular endothelium, it has been proposed that kidney cells might be susceptible to direct SARS-CoV-2 infection [26,27], and infectious virus has been successfully isolated from urine to support this claim [267]. The observed renal damage in COVID-19 can probably be attributed to indirect mechanisms: increased inflammation (which is amplified by CKD), hemodynamic instability, rhabdomyolysis, microthrombi and hypoxia are plausible causes of acute kidney injury (AKI) [268].

The possible contribution of liver diseases to the risk of severe COVID-19 [208,280,281] might be related to the M1 polarization of macrophages causing an increased level of systemic inflammation [386]. However, it has been pointed out that liver diseases often co-occur with other important NCDs, and may not have an independent effect on COVID-19 [387]. Liver injury during COVID-19 might be a result of direct infection [26,388], immune-mediated inflammation [389] and/or antiviral medication use [390,391].

Cancer patients tend to be old, comorbid and immunocompromised in a variety of ways [209,392,393], which may influence COVID-19 outcomes, but certain malignancies are likely to have also a direct impact [394]. The risk of hospitalization and death with COVID-19 have both been associated with cancer [201,208,209,395]. Hematologic [280,281,395,396] and lung cancers [397], and cancers with advanced/metastatic stages [397,398] may have the strongest effect. In addition to the direct effects of cancer, anticancer therapy (chemotherapy, immunotherapy, radiotherapy) might also influence COVID-19 outcomes [394]. However, current limited evidence does not support a strong effect on disease severity [397,399,400].

Both immune-mediated inflammatory diseases [311] (e.g., rheumatoid arthritis [201]) which are characterized by dysfunctional cytokine responses, and the use of immunosuppressant medications [13,280], have been associated with poor COVID-19 outcomes. Special attention has been given to glucocorticoids [319,321,401,402]. While dexamethasone has been demonstrated to reduce mortality with severe/critical COVID-19 [403], it has also been shown that chronic (and especially high-dose) intake of glucocorticoids, and use in mild cases are connected to increased hospitalization and mortality [401]. This effect is likely mediated by the suppression of IFN responses and antimicrobial peptide secretion (causing respiratory dysbiosis) [404,405]. Patients who receive immunosuppressive drugs after organ transplantation are also at a higher risk of severe outcomes during COVID-19 [201,269,280]. Primarily immunocompromised individuals may similarly face poor COVID-19 outcomes (see Section 2.2. *Human Genetic Variation*); in this group, chronic lung diseases, insufficient vaccine responses and, in the most prevalent subgroup (common variable immunodeficiency), T- and B cell dysfunctions are commonly observed [406].

Some neurological conditions have also been linked to COVID-19 severity [201,280,407]. Possible mechanisms include immunosenescence, heightened IFN responses or genetic predisposition to severe COVID-19 (OAS1, APOE ε4 allele) in Alzheimer’s disease [350,408,409,410], respiratory muscle rigidity and insufficient cough reflex in Parkinson’s disease [350,411], systemic inflammation in epilepsy (with much uncertainty) [362,412] and susceptibility to acute stress in cerebrovascular diseases [413,414]. Increased levels of chronic inflammatory mediators have been observed in several mental disorders (major depressive disorder, bipolar disorder, schizophrenia, and sleeping disorders) [415].

We conclude that some common chronic comorbidities appear to have a strong impact on the risk of severe COVID-19 outcomes, while other conditions have weaker or less clear effect on the course of infection.

#### 2.4.2. Coinfections/Superinfections

Coinfection refers to the simultaneous infection of a host by two or more pathogen species or strains, while superinfection is the acquisition of a second infection after, and in addition to, the first. In both cases, the simultaneous presence of two pathogens can modulate—exacerbate or ameliorate—the effects of either or both. Of note, exacerbating interactions have had a substantial impact on past influenza pandemics, where most deaths were often caused by secondary bacterial infections [416,417,418].

Co- and superinfections are often hard to distinguish in clinical settings (due to limitations in sample collection and pathogen identification, and the lack of clear definitions), and most studies on SARS-CoV-2 did not differentiate between the two scenarios [419]. According to a meta-analysis, in the studies that did distinguish between the two forms of dual infection, the overall rate of superinfections (19–30%) was slightly higher than the prevalence of coinfections (14–25%) [420]; most studies reported coinfections and superinfections among hospitalized cases.

In Table 5, we compiled the results of studies that tested the association between coinfection with specific pathogens and the severity of COVID-19, hypothetical causative mechanisms for the associations, and potential mechanisms by which a pre-existing infection might facilitate the acquisition of SARS-CoV-2 (or vice versa). Data about coinfections were not available beyond case reports or case series for dengue virus [421], certain human herpesviruses [422] (cytomegalovirus [423], Epstein–Barr virus, human herpesvirus 6), fungi causing mucormycosis [424] and *Pneumocystis jirovecii* [425], and we therefore did not include detailed information on these pathogens in the table. We also note that the effect of some pathogens on COVID-19 severity is highly debated and may depend on the severity and the degree of clinical control of the coinfections.

Infection by SARS-CoV-2 might also facilitate co- or superinfections with some pathogens. Enhanced adherence to infected cell lines [530,531,532], reduced ciliary function and clearance [533,534], altered mucus secretion (goblet cell hyperplasia) [535,536], reduced oxygen exchange [537,538,539], virus-induced [533] and immune-mediated (e.g., by NETs) [540] cytotoxic airway damage, disruption of innate immunity followed by hyperinflammation [541], immunosuppressive effects of platelet activation [542], decreased levels of adaptive immune cells [35,543] and induced microbiota dysbiosis (both respiratory and gastrointestinal through the gut–lung axis) [426,544] are all possible mechanisms. In turn, some other acute infections might promote superinfection with SARS-CoV-2 through similar mechanisms; however, we are not aware of any studies designed to test this effect. Finally, in the context of long-term chronic infections, superinfection with SARS-CoV-2 (over a pre-existing condition) may be more likely than acquiring the other pathogen during the brief course of a COVID-19 episode. Uncontrolled HIV infection, in particular, appears to increase susceptibility to SARS-CoV-2 [468], and to promote persistent COVID-19 in some patients [545], by suppressing efficient immunity. Certain chronic coinfections predispose to NCDs as well (see Section 5.1. *Interactions Between Risk Factors of Severe COVID-19*). Furthermore, SARS-CoV-2 infection has been reportedly connected to the reactivation of latent hepatitis virus [546] and *Mycobacterium tuberculosis* infections [442,547].

Recent meta-analyses estimate the prevalence of bacterial co- or superinfections at approximately 15–20% among hospitalized cases [548,549] with an even higher prevalence in severe cases [430,549,550,551]. While (community-acquired) bacterial coinfections seem to be relatively rare (3–8%) [420,430,550] even compared to RSV or influenza virus patients [434], (hospital-acquired) secondary infections are quite common (14–24%) [419,420,550]. At hospital admission, *Klebsiella pneumoniae*, *Streptococcus pneumoniae*, *Staphylococcus aureus* and *Haemophilus influenzae* were the most frequently detected coinfecting species, while superinfections in the hospital were typically caused by *Acinetobacter* spp., *Pseudomonas aeruginosa*, *Escherichia coli*, *Staphylococcus aureus* and *Klebsiella* spp. [420,430]. Unfortunately, most studies of bacterial superinfections had low sample sizes, which resulted in very wide margins for the effect sizes and indicates a low certainty of demonstrated effects.

The overall prevalence of viral infections detected concurrently or following COVID-19 is approximately 7–12% [548,549,552] with a higher rate of coinfections (5–10%) [420,553] compared to superinfections (~4%) [420]. The probability of acquiring a viral superinfection during COVID-19 may be lower than observed in the general population [554], and there were no differences found in prevalence between cohorts of severe and mild cases [549]. Common coinfecting viruses are influenza viruses, RSV and enteroviruses (particularly, rhinoviruses) [420,553]. Epstein–Barr virus (EBV), human herpesviruses (HHV), seasonal human coronaviruses (HCoV), adenoviruses and human metapneumovirus (HMPV) were also reported in several studies [490,549,552,555,556]. The overall effect of viral co/superinfections on the severity of COVID-19 is unclear [550,553,556,557], but the common phenomenon of viral interference [558], and differences between the relative proportion and effect of specific pathogens in the studies may be responsible for these mixed results.

Fungal superinfections are more common (4–13%) [420] than coinfections (2–7%) [420] and both are connected to increased mortality [431,559]. Invasive pulmonary aspergillosis (*Aspergillus* spp.) is dominantly present [420,549,551], especially among coinfections [420], while invasive candidiasis (*Candida* spp.) is the most frequent complication among superinfected cases [420]. Mucormycosis (*Rhizopus* spp., *Mucor* spp.etc.) [424] and pneumocystis pneumonia (*Pneumocystis jirovecii*) [425,560] have also been reported repeatedly. Individuals with DM or patients receiving corticosteroid treatment have a higher risk of severe SARS-CoV-2–fungal coinfections (aspergillosis and mucormycosis) [509,561,562].

The prevalence of parasitic coinfections is strongly heterogeneous geographically and has not been assessed systematically in COVID-19 patients. In an Ethiopian cohort study, *Entamoeba* spp. (~20%) and *Giardia* spp. (~4%) were common parasitic protozoa, while *Hymenolepis nana* (~17%), *Schistosoma mansoni* (~5%) and *Ascaris lumbricoides* (~4%) were commonly identified helminth species [519]. In Egypt, *Toxoplasma gondii* (~22%), *Cryptosporidium* spp. (~20%), *Blastocystis* spp. (~17.6%) and *Giardia* spp. (~9%) were reported [563]. In Sub-Saharan Africa and South Asia, a meta-analysis estimated the overall prevalence of *Plasmodium* spp. approximately 11% among COVID-19 patients [564]. In Brazil, COVID-19 patients coinfected with Chagas disease (*Trypanosoma* spp.) were rare (~0.4%) [528].

Finally, we note that in the study of associations between co/superinfections and COVID-19 severity, causality is often hard to establish. In particular, individuals with coinfections might be more likely to have weakened immunity or general health, which would imply a hidden shared common cause for the coinfection and subsequent severe COVID-19, rather than direct causality.

### 2.5. Frailty

Frailty is a medical condition affecting multiple organ systems, characterized by reduced strength and endurance, impaired physiologic and immunological functions, and a reduced ability to combat acute stressors, leading to increased dependency and/or death [565,566,567]. The prevalence of frailty is estimated to be approximately 5–9% worldwide over the age of 50, but it varies considerably by demographic variables (e.g., age and sex) and also geographically (higher in low- or middle-income countries in spite of younger populations) [568], with notably higher rates among nursing home residents (~50% over the age of 60) [569] compared to community-dwelling individuals (~10% among people older than 65) [570]. Importantly, frailty is strongly linked to, but is not equivalent to, aging, and it is typically quantified by the Clinical Frailty Scale (CFS) from 1 (very fit) to 9 (terminally ill), which is a composite marker based on a clinical assessment of physical ability, comorbidity, cognitive impairment, and disability [571].

The effect of frailty on in-hospital COVID-19 mortality has been demonstrated in large clinical studies [572,573,574,575], and confirmed by meta-analyses [576,577]. Mildly frail patients (CFS 4–5) had increased risk of severe outcomes compared to fit individuals (CFS 1–3) in several (but not all) clinical studies [573,576]; severe frailty (CFS 6–9) showed a correlation with severe disease consistently and with a greater effect size [572,573,578]. COVID-19 mortality is gradually increasing with the CFS even when controlling for age and sex [579,580]. A similar relationship has been observed with all-cause mortality and different frailty measures as well [581,582,583].

The development of frailty has been linked to chronic inflammation, which is a major factor in the pathogenesis of severe COVID-19 [584,585]. Both conditions share inflammatory and immunological biomarkers (IL-6, CRP, LDH, PCT, and cortisol) [584] and lead to heightened coagulation, development of sarcopenia and decline in multi-system function [586].

In summary, although frailty syndrome is a biologically overlapping condition with aging and multimorbidity, it appears to also have an independent effect on the outcome of COVID-19, and it is an important predictor of disease severity upon hospital admission.

### 2.6. Microbiota

SARS-CoV-2 infection is often accompanied by respiratory and intestinal dysbiosis with characteristic patterns that are distinguishable from those induced by influenza infection [587] or other forms of community-acquired pneumonia [426,588]. While most of these changes can attributed to the presence of SARS-CoV-2 and host–virus interactions, some evidence supports a bidirectional relationship between the composition of the microbiota and COVID-19 disease severity, implying that pre-infection variation in the microbiota might influence clinical outcomes. Gastrointestinal microbiota composition has a complex relationship with several COVID-19 risk factors, such as age, NCDs, lifestyle and the frailty syndrome (for details see Section 5.1. *Interactions Between Risk Factors of Severe COVID-19*), indicating at least a mediator role in the determination of severe COVID-19.

Probiotics inhibit the growth of pathogenic microorganisms [589], enhance immune responses [590], and have been effectively used in the treatment of metabolic diseases (obesity and DM) [591]. As a therapeutic option in the management of COVID-19 probiotic treatments have yielded limited and mixed results [592,593]. Antibiotics constrain the growth of opportunistic pathogens and can prevent secondary infections, but they also perturb the healthy microbiota. This disruption of the microbial communities might have an impact on COVID-19 severity. Individuals with repeated recent exposure to antibiotic treatment have been shown to have increased severity of disease [594,595], although this might have a causal relationship with the original cause that necessitated treatment, rather than with the treatment itself. Those who received antibiotics during early (non-severe) stages of COVID-19 subsequently had increased severity and stayed longer in hospital [596], but did not have increased mortality [596]. Altogether, several lines of indirect evidence indicate that the composition and diversity of the human microbiota might play a role in the determination of COVID-19 severity, possibly independently from other COVID-19 risk factors.

The effect of the microbiota on clinical outcomes is likely to be mediated by its response to SARS-CoV-2 infection. In addition to other important functions, the microbiota are responsible for promoting and maintaining a stable and immunologically stimulating environment both in the respiratory and gastrointestinal tracts [597,598]. The disruption of the microbiota involves several processes that might contribute to COVID-19 pathogenesis; conversely, microbiota that is resilient to the effects of COVID-19 may have a protective effect.

SARS-CoV-2 replication initiates in the URT and continues in the alveoli of the lungs influencing the local immunological environment. This causes characteristic alterations in the diversity and composition of the microbiota in the oral cavity [599,600] and the upper [426,601] and lower respiratory tract (LRT) [602,603]. The diversity of the microbiota typically increases in the URT [604,605,606] due to the emergence of opportunistic pathogens (*Klebsiella*, *Streptococcus*, *Veillonella*, *Prevotella*, *Enterococcus*, *Rothia*, etc.). Consequently, local inflammation increases in the oral cavity (IL-6, IL-17) [605], the URT (IL-6, IL-8, IL-1β) [607] and the lungs (white blood cell (WBC) and lymphocyte (LYM) counts) [544] together with changes in the host metabolic profile in the RT (reduced protein, lipid and glycan metabolism, induced nucleotide and amino acid biosynthesis and carbohydrate metabolism) [588,606].

Pro-inflammatory cytokines from the lungs are transferred to the circulatory system [608], which then induces changes in the gastrointestinal microbiota (gut–lung axis) as has been shown in influenza infections [609,610,611]. This connection is confirmed by the fact that COVID-19 is often accompanied by GI symptoms [612]. These affect approximately 5–10% of patients (most often anorexia, nausea, vomiting, and diarrhea) [613,614], and might persist long after viral clearance [615]. As in the RT, the proportion of anti-inflammatory probiotic and beneficial commensal bacteria (*Lactobacillus*, *Bifidobacterium*, *Eubacterium*, *Faecalibacterium*, *Roseburia*, *Lachnospiraceae*, etc.) typically decreases, while opportunistic pathogens and pro-inflammatory species (*Streptococcus*, *Veillonella*, *Actinomyces*, *Clostridium*, *Bacteroides*, etc.) expand in the GIT during COVID-19 [616]. Contrary to the RT, microbial diversity declines in the intestines [587,617] due to the depletion of rich commensal communities. Unique alterations occur to the fungal (higher levels of *Candida*, *Aspergillus*, *Auris*, etc.) [600,617], viral [602,606] and archaeal [606] communities as well both in the RT and the GIT. The microbial diversity in most cases quickly returns to normal values after recovery [618] and the magnitude of dysbiosis is proportional to certain immunological and metabolic signatures of COVID-19 and severity of disease [544,619,620].

In line with compositional changes in the microbiota, altered biosynthetic and metabolic pathways, including more intense vitamin B12 and urea production along with impaired short chain fatty acid (SCFA), L-isoleucine, tryptophan and polyamine biosynthesis and sulfur oxidation were typical in SARS-CoV-2-infected patients [618,621,622,623,624]. Some of these metabolic signatures seem to show sexual dimorphism in COVID-19 patients compared to uninfected controls [625]. Rise in inflammatory molecules in the GIT has also been shown during COVID-19 (i.e., CRP, PCT, D-dimer, LDH, AAT, and GGT) [587,626]. In particular, fecal butyrate levels were found to be negatively associated with some of these biomarkers (IL-10, CXCL-10, and CRP), similarly to L-isoleucine (CXCL-10) [622], SCFAs [627] and derivatives of bile acids [628], which have broad effects on the immune system. Butyrate in particular has important roles in the induction of Treg cells [629,630], it reduces several pro-inflammatory pathways [631], participates in the secretion of mucins and defensins [629] and helps to maintain the intestinal barrier [632]. Causal connections between beneficial and harmful microbes, inflammation and metabolic responses have been established by multi-omics analyses [620,633].

Local inflammation and dysbiosis damage the integrity and increase the permeability of the intestinal barrier [623,634], which might facilitate the infection of intestinal epithelial cells by SARS-CoV-2 [635]. In turn, direct infection and viral replication might exacerbate dysbiosis in different ways. First, decline in intestinal ACE2 levels by infection and/or the loss of beneficial bacteria [636] might aggravate inflammation through the RAS [637]. As an alternative mechanism, reduction in ACE2 might also downregulate the amino acid transporter B0AT1 (heterodimer formation with ACE2 [638]), which is responsible for tryptophan absorption [639]. With reduced levels of tryptophan the secretion of antimicrobial peptides decreases [639,640], which in turn aggravates dysbiosis. Severe dysbiosis and increased intestinal permeability might lead to the translocation of pathogens, toxins and cytokines to the circulatory system leading to severe complications and multi-organ failure [641]. The causal role of COVID-19-induced gastrointestinal dysbiosis in the development of symptoms and disease severity is further supported by results obtained with a gnotobiotic mouse model [642]. Fecal microbiota transplantation from COVID-19 patients to germ-free mice resulted in lung histopathology, an inflammatory cytokine profile, cognitive impairment, and increased susceptibility towards bacterial infection in the animal model indicating that pre-infection differences in microbiota composition might influence COVID-19 susceptibility and severity as well. Further studies on mice suggest that gut dysbiosis also damages the blood–brain barrier [643,644], induces neuroinflammation [644,645] and facilitates direct neuroinvasion by SARS-CoV-2 [646].

To conclude, microbial dysbiosis is a characteristic trait of SARS-CoV-2 infection that may be both cause and consequence in the pathogenesis of COVID-19. Multiple factors associated with an altered microbiota have been connected to COVID-19 severity. However, further studies are needed to explore the causal relationships between the microbiota and COVID-19 pathogenesis, controlling for the interrelated effects of age, lifestyle, and comorbidities.

### 2.7. Immunological History

#### 2.7.1. Previous SARS-CoV-2 Infection

SARS-CoV-2 infection elicits both cellular and humoral immunity, which strongly reduces the risk of severe clinical outcome in subsequent re-infections, and provides partial protection against re-infection (reviewed in [647,648]).

Following successful immunization, B cells (and antibodies) are thought to be responsible for SARS-CoV-2 inoculum neutralization, early control and inhibition of viral replication, while T cells are mainly the agents of cellular control of infection in addition to their role in the coordination of immune responses [648]. For this reason, humoral immunity might be effective against both reinfection and severity of disease, while cellular immunity mainly reduces severity of COVID-19 [649]. Regarding the molecular targets, cellular responses target mainly structural proteins (S, M, N) of SARS-CoV-2, but some CD4+ and CD8+ T cells recognize accessory and non-structural proteins as well [650]. Similarly, antibodies mainly target epitopes on the S and N proteins as potential targets of neutralization [651].

Following SARS-CoV-2 infection the diversity and affinity of antibodies keeps increasing for several months [652] along with the level of memory B cells [648], but the levels of most immune components (IgG, IgA, and T cells) decline exponentially even in the first month post infection [648]. In the absence of a new variant with substantial immune evasion capabilities (such as Omicron variants), natural immunity might retain its protective effect for 8–12 months against reinfection [647,652,653,654,655] and probably longer against severe manifestations [648]. This is consistent with the observation that relatively low antibody titers show 50% protective effect against symptomatic and severe disease (14.4–28.4% and 0.71–13% of the initial magnitude, respectively) [656]. In addition to immunoglobulin levels, the persistence of peripheral SARS-CoV-2-specific CD4+ and CD8+ T cells is also a determinant of effective immune protection against reinfection and disease control [657,658].

While a detailed discussion of the impact of vaccinations (and other medical interventions) on COVID-19 outcomes goes beyond the scope of this review, we note that there are some differences between vaccination and natural infection in the presentation of antigens and the qualities of the developing immune memory. While vaccination induces systemic immunity, and the most widely used vaccines elicit only spike-specific humoral and cellular immunity, natural infection generates immunity against all viral proteins, and induces tissue-specific (e.g., mucosal) responses as well [648]. However, some vaccines evoke higher IgG levels compared to natural infection (regardless of severity) [659,660]. Short-term immunity after infection and vaccination might be similarly effective [661], but the duration of protection seem to be significantly shorter in the latter case (~6 months for reinfections [647] compared to the previously mentioned 8–12 months following SARS-CoV-2 infection), possibly due to the more rapid decay of antibody titers [659]. This might also explain why in real-world settings reinfection results in less severe disease compared to COVID-19 following vaccination [662]. Nonetheless, this distinction is probably losing its importance as the pandemic progresses and increasing numbers of individuals accumulate exposure to both vaccination and infection, developing ‘hybrid immunity’. It has been shown repeatedly that individuals with hybrid immunity can acquire stronger neutralizing antibody levels compared to individuals with vaccination induced or natural immunity alone [659,663].

In the first two years of the pandemic, reinfections were rare (<2% of followed cases) [664,665,666] confirming the protective effect of specific immunity. Additionally, the severity of reinfections were significantly lower compared to primary infections (aOR = 0.10 (0.03–0.25) [667], aOR = 0.39 (0.35–0.44) [668]). At the end of 2021, with the rise of the Omicron variant, which showed similar transmissibility but more effective immune evasion compared to previous variants of concern (VOCs) [669], the rate of reinfections rose significantly [670,671,672]. However, those individuals who had had prior immunity were less infectious compared to immunologically naïve individuals [673] indicating lower viral burden and better disease control. Then, in 2022, repeated waves of Omicron subvariants were consistently characterized by the lack of evidence for (re-)increased severity of disease compared with the preceding wave [674,675], which indicates long-lasting protection against severe disease, while immune escape mutations appear to be able to erode protection against re-infection rapidly.

COVID-19 disease severity is modulated by additional factors even in the presence of immunological protection resulting from previous SARS-CoV-2 infection(s). Advanced age [668,676,677], the presence of comorbidities [676,677,678,679] and male sex [668] have an exacerbating effect on re-infections similar to first infections. Severe primary infection predicts higher risk of severe symptoms in re-infections [676,677], even though more severe primary infection appears to elicit higher levels of memory B cells [680] and antibodies [680,681,682,683], and stronger T cell responses [650]. A higher severity of reinfections is associated also with markers of dampened immune protection (low avidity IgG [684], and longer time between infections [678]).

In summary, previous SARS-CoV-2 infection provides substantial protection against severe COVID-19 in subsequent re-infections, possibly modulated by, but largely independent of other risk factors, and this protection appears to last longer and be more robust to viral evolution than the protection against re-infection.

#### 2.7.2. Cross-Reactive Immunity from Other Infections

Some evidence indicates that immune responses elicited by previous, non-SARS-CoV-2 infections might also influence the outcome of COVID-19, if pre-existing immune responses can cross-react to SARS-CoV-2 epitopes. Based on sequence similarity, cross-reactive responses to SARS-CoV-2 are most likely to involve pre-existing immunity to other human coronaviruses (HCoV), of which two betacoronaviruses (HCoV-229E, HCoV-NL63) and two alphacoronaviruses (HCoV-OC43, HCoV-HKU1) are responsible for an estimated 10–15% of common cold episodes [685]. However, pre-existing cross-reactive immunity to SARS-CoV-2 may not be entirely explained by previous exposure to HCoVs [686,687], and may include responses to unrelated infections such as influenza or cytomegalovirus infections as well [688].

Of the distinct arms of adaptive immunity, CD4+ T cell responses against epitopes conserved across SARS-CoV-2 and other coronaviruses are present in COVID-19 convalescent patients [650,689,690,691] and more importantly, often in unexposed healthy individuals [689,692,693,694,695] as well, with proportions up to 50–80% in some of the unexposed populations analyzed [650,690,696]. Antigenic targets consist of structural (S, N, M) [650,689,690,695], non-structural [650,690] and accessory proteins [650,690]. These pre-existing T cells seem to cross-react with both SARS-CoV [650] and all four common cold coronaviruses [692]. Preexisting CD8+ T cells showed similar cross-reactivity [688,697,698,699,700]. While a correlation between the level of pre-existing cross-reactive T cells and clinical severity during COVID-19 has (to our knowledge) not been tested directly yet, potential effects on the course of the disease include the priming of protective immunity [701] and contributing to an early control of viral replication [702]. However, these T cells often have low avidity against SARS-CoV-2 antigens, and de novo immune responses are probably required for effective control of SARS-CoV-2 replication [703].

Humoral immune responses from previous infections might also display cross-reactivity against SARS-CoV-2. SARS-CoV-2 cross-reacting antibodies have been detected in several [704,705,706,707], but not all [708,709,710] studies that analyzed sera obtained or intravenous immunoglobulin manufactured before the pandemic. Some studies found cross-reactive antibodies in only a small percentage of individuals [27,28], implying that genuine variability might have existed within or between populations, possibly related to differences in infection history. The most likely source of SARS-CoV-2 cross-reactive antibodies are HCoV-specific memory B cells [711]. The molecular targets are mainly the S [704,705,711,712,713] and N proteins [694,704,705,713,714], but cross-recognition of conserved non-structural proteins [704] has also been reported. These antibodies recognize SARS and MERS coronaviruses [712] along with seasonal HCoV antigens [694,711,712,714,715,716], indicating broad cross-reactivity. Due to closer relatedness, the probability to cross-react to SARS-CoV-2 might be higher for immune responses that had been elicited against HCoVs belonging to the betacoronaviruses compared to those that targeted earlier alphacoronavirus infections. However, this prediction has not been tested by a systematic comparison, and while some studies suggest a primary role of betacoronavirus cross-immunity [711,712], others do not appear to support the hypothesis [694,707,713,716]. In addition, in COVID-19 convalescent individuals the levels of seasonal HCoV cross-reacting antibodies are typically also boosted, which provides further support for cross-reactivity. However, while some studies demonstrated this effect for antibody levels against all seasonal HCoVs [717,718,719], in others the effect was limited to titers against betacoronaviruses [720,721,722,723] (especially HCoV-OC43 [707,724,725,726,727]), or surprisingly, to alphacoronaviruses only [728,729]. Independent of the level of antibodies, SARS-CoV-2-specific IgG and IgM were shown to robustly recognize betacoronavirus antigens [712,715,730].

Several studies have investigated the potential impact of pre-existing HCoV-specific antibodies on COVID-19 severity; unfortunately, the conflicting results do not allow a firm conclusion. Some analyses demonstrated a correlation between HCoV-specific antibody titers and milder COVID-19 outcomes [718,728,729,731,732,733,734,735], other studies found no significant effect [709,736,737], and some studies reported a correlation between cross-reactive antibody titers and more severe COVID-19 outcomes [707,720,722,723,726,738]. A beneficial effect might be explained by effective cross-neutralization or the priming of effective humoral immune responses, while a negative effect might arise from low-avidity cross-reactive immunoglobulins hindering the production of high-avidity SARS-CoV-2-specific antibodies. Antibody-dependent enhancement of SARS-CoV-2 has also been proposed [739], but not confirmed.

It must be noted that cross-reactivity does not necessarily, or even typically, imply cross-neutralization against SARS-CoV-2. Neutralizing antibodies have been described in some studies [740,741,742], but most analyses have failed to demonstrate neutralization activity [707,709,722,743,744,745].

We conclude that while the existence of cross-reactive immunity to SARS-CoV-2 has been demonstrated by several studies, the impact of this immunity on COVID-19 outcomes remains largely hypothetical. Finally, we note that any impact of cross-reactive immunity to other infections is likely to have been restricted to the first waves of the pandemic, and immunity to SARS-CoV-2 in subsequent waves has probably been dominated by specific immunity from previous episodes of COVID-19.

### 2.8. Lifestyle

#### 2.8.1. Physical Activity

Regular physical activity appears to have a beneficial effect on the outcome of COVID-19. Individuals living a sedentary lifestyle are exposed to a higher risk of COVID-19 hospitalization (OR = 2.26 (1.81–2.83)) [746], ICU admission (OR = 1.73 (1.18–2.55)) [746], severe disease [747] and mortality (OR = 2.49 (1.33–4.67)) [746,747,748,749] compared to individuals who exercise regularly. In detailed analyses, higher metabolic equivalent of task per week was associated with a lower risk of COVID-19 hospitalization [750], severe disease [747] and mortality [747,749]. Similarly, cardiorespiratory fitness was also correlated with the severity of disease [751] and the risk of death [752]. The beneficial effects of physical training seem to be long-lasting. Among male military conscripts, high cardiorespiratory fitness and muscle strength in late adolescence and early adulthood proved to be protective against the adverse effects of COVID-19 decades later [753].

Regular physical activity has broad effects on human metabolism and the immune system that might be protective against severe COVID-19. An active lifestyle has been linked to lower incidence and severity of URT viral infections (e.g., influenza) [754,755] in humans, and to attenuated inflammation following bacterial infection in mice [756]. Both aerobic and muscle strength training stimulate the release of myokines (e.g., myostatin, IL-6, IL-15, and LIF) [757], which in the long term counteract low-grade chronic inflammation [758]. Exercising can also boost innate [759] and adaptive immune responses [760,761,762], and helps to maintain local tissue immunity [763] (e.g., in the lungs [756]) and to delay immunosenescence [764]. In addition to immunological functions, regular exercising helps to slow down the deterioration of frailty by preserving muscle [765] and respiratory function [766], and prevents body fat accumulation [767] and the development of CVD [768]. Loss of adipose tissue lowers the leptin/adiponectin ratio and hence, chronic inflammation [769].

The impact of physical activity on COVID-19 may thus be mediated by its effects on immunity, comorbidities, and frailty. For further details on the effects of physical activity on COVID-19 pathogenesis, we recommend reading the review by Filgueira et al. [770].

#### 2.8.2. Alcohol Consumption

High consumption of alcohol has been linked to adverse health effects, including an exacerbation of *Mycobacterium tuberculosis* infection and other sources of ARDS [771,772]. While some early reports failed to find a significant effect of alcohol consumption on COVID-19 severity [773,774,775], excess alcohol intake has since been repeatedly associated with worse clinical outcomes, such as severe disease [776], ARDS [777] and death [778]. A latent causal variable analysis that considered also genetic correlations using GWAS data also confirmed the link between alcohol consumption and severe COVID-19 [779]. Furthermore, as with many other risk factors, alcohol consumption has been shown to correlate with the level of proinflammatory biomarkers (e.g., CRP and NLR) [778] and proinflammatory cytokines (IL-1β, IL-6, and TNF-α) [780,781]. It causes oxidative stress [782], impacts the activity of alveolar macrophages [781,782], T lymphocyte proliferation and turnover [781] and the number and function of NK cells [783]. Alcohol use in the long term also has an impact on the endothelial cilia and respiratory clearance [784]. Alcohol-related liver disease has also been shown to increase the risk of COVID-19 mortality [310].

#### 2.8.3. Smoking

The consumption of combusted tobacco products has long been known to have a detrimental effect on lung function and health. In accordance, smoking might induce more severe lung inflammation and respiratory distress during COVID-19, similarly to influenza virus infections [785]. However, the results of association studies in the case of COVID-19 have been somewhat controversial. Some analyses that considered smoking habits [786] and a genetically predicted tendency to smoke [787,788] have been linked to more adverse outcomes in COVID-19 patients. However, the frequency and duration of smoking, or time since quitting tobacco use also influence the increased risk posed by this habit. The long-term damaging effects of smoking have been studied by comparing never-smokers to former [204,789,790,791,792] or ever-smokers (former and current smokers combined) [124,204,793,794,795], which consistently showed more severe outcomes (e.g., hospitalization, ICU admission, MV and death) in the latter groups. However, although current smokers compared to (current) non-smokers seem to be more prone to experience severe symptoms and death [123,794,796,797], when comparing current smokers to never-smokers, some publications reported increased severity [204,788,796,798], while others no effect [791,792,799], and surprisingly, recent cohort studies found lower rates of severe outcomes [789,790] in the smoker group of the study. The short-term effects of tobacco use are harder to measure in clinical settings, but ambiguous results on the effect of current smoking behavior on COVID-19 might imply further physiological and immunological effects that differ from the mechanisms responsible for long-lasting damage in the lungs.

Several components of combusted tobacco products show immunomodulatory (e.g., polycyclic aromatic hydrocarbons (PAHs), acrolein, and CO) and/or harmful effect (e.g., volatile organic compounds, metals, oxidants, and nicotine) on human health [800]. These facilitate the development of chronic lung disease [801], CVD [802] and DM [803], which might confound the estimation of the direct effect of smoking if not controlled for properly. Possible direct effects of smoking on COVID-19 severity include impairment of mucociliary clearance [804], increased epithelial permeability [805], immune suppression [806] (IFN responses in particular [807]), elevated oxidative stress, inflammation and vascular injury [808,809]. Potential mechanisms proposed to explain a beneficial effect of current smoking demonstrated in some studies include the modulation of the RAS by increased ACE2 levels in the lungs of smokers [810,811], increased NO inhibiting viral replication [812], or inhibition of pro-inflammatory cytokine secretion [813,814] by nicotine.

We conclude that the effect of smoking on COVID-19 appears to be complicated and requires further study. The review by Benowitz et al. [800] provides a more detailed discussion of the topic.

#### 2.8.4. Diet and Nutrition

Dietary habits influence metabolism and the risk of developing chronic diseases. Malnutrition can result in a wide range of unfavorable health effects [815], including impaired immunity [816], and is independently associated with mortality among older adults [817]. COVID-19 patients who experienced malnutrition either years before SARS-CoV-2 infection [818,819] or during COVID-19 [820,821] were more likely to experience adverse outcomes, such as prolonged hospitalization, MV or mortality. Among those considered well nourished, food choice may still have some influence on the severity of COVID-19. Some studies have reported that high-quality (defined by multiple measures) [822], vegetarian [823] or plant-based [824,825] diets were associated with more favorable disease outcomes. Adherence to the Mediterranean diet (high in fruits, vegetables, legumes, olive oil, and whole grains; low intake of processed foods and red meat), which involves a high intake of antioxidants [826], boosts immunity and reduces inflammation [827], was also associated with less severe COVID-19 in small cohorts [828,829]. Preliminary evidence supports that other anti-inflammatory dietary patterns such as intermittent fasting [830] and ketogenic diet [831,832] might be beneficial.

No single food item has been unambiguously connected to COVID-19 severity [833]. However, certain metabolic biomarkers, including essential nutrients, are useful biomarkers of COVID-19 outcome [834]. Current research focuses mostly on two nutrients, omega-3 polyunsaturated fatty acids (PUFAs) and vitamin D.

Although sample sizes were small, some studies indicate that regular consumption of omega-3 PUFAs might be favorable against severe COVID-19. Low omega-3 index [835] and omega-3 PUFA deficiency [836] have both been associated with adverse outcomes, such as MV and mortality. Similarly, omega-3 PUFA supplementation has been connected to milder symptoms [837,838], and omega-3 supplementation had previously been shown to improve symptoms in ARDS patients [839]. These effects might be mediated by the role of omega-3 PUFAs in the enzymatic conversion of specialized pro-resolving mediators (SPMs) [840]. SPMs play an important role in the termination of inflammatory reactions by preventing the infiltration of phagocytes, enhancing the removal of apoptotic cells and debris, inhibiting cytokine production and removing inflammatory mediators [841]. Omega-3 supplementation increases SPM levels [842], which also correlate with mild COVID-19 [843]. Lastly, certain PUFAs (such as the omega-3 eicosapentaenoic acid) have been shown to interfere with the binding of the SARS-CoV-2 RBD to hACE2, and to TMPRSS2 and CTSL in vitro [844].

The active form of vitamin D, 1,25(OH)2D has important functions in innate antiviral immunity against several respiratory viruses, such as rhinoviruses [845], RSV [846] and influenza viruses [847]. 1,25(OH)2D induces the production of antiviral effector molecules, enhances the activity of innate immune cells and Tregs, and also lowers TNFα, Th1 and Th17 cell levels, and the ACE2:ACE ratio [848,849]. In COVID-19, some studies have found an association between lower levels of the major circulating form of vitamin D, 25(OH)D, and an increased severity of COVID-19 [850,851,852]. However, as has been pointed out elsewhere [848], these results may indicate either worse COVID-19 outcomes due to low initial 25(OH)D levels [848], or, conversely, they might reflect relevant metabolic changes as a result of more severe disease [853]. A recent review by Martineau and Cantorna [848] reported that out of 11 randomized controlled trials that had been published at the time of writing, only 4 reported significant associations. Two recent large studies and a systematic review on vitamin D supplementation reported lower severity and mortality in the treated groups compared to controls [854,855,856], a study using Mendelian randomization found no association between genetically predicted 25(OH)D levels and COVID-19 severity [857], and another genetic analysis demonstrated an association between a risk score constructed from several genetic variants that influence vitamin D pathways and with 25(OH)D levels, and between 25(OH)D levels with disease outcome [858]. Recently, a mechanistic link has been proposed between vitamin D supplementation and reduced COVID-19 severity through the increased expression of interferon stimulating genes and higher protein levels both in vitro and in vivo [859].

We conclude that more studies are needed to clarify the importance and magnitude of the effect of Omega-3 PUFAs and vitamin D on COVID-19. For further details, we recommend reading the reviews by Mazidimoradi et al. [836], and by Martineau and Cantorna [848], respectively.

## 3. Viral Factors

### 3.1. Viral Genetic Variation

Viral genetic factors have long been known to influence the outcome of infection in other well-studied viral epidemics [860], and it has quickly become clear that the emerging variants of SARS-CoV-2 responsible for successive waves of the epidemic (designated Variants of Concern, VOCs) can influence not just the transmissibility of the virus, but the severity of COVID-19 as well (Table 6). Because the risk of severe outcome is influenced by multiple factors that can differ between countries and even between successive waves, risk ratios of severe disease could be reliably estimated when two variants were simultaneously present (typically: one replacing the other) in the same country or region.

The first marked effect was observed when the Alpha VOC (Pango designation: B.1.1.7) replaced basal non-VOC variants in the UK in late 2020, then spread to become the dominant lineage worldwide in the first half of 2021. Two large studies using S gene target failure as a proxy for Alpha infection estimated that the risk of hospitalization and death was approximately 64% and 55% higher in Alpha, compared with non-VOC infections [861,862].

Limited data are available on the severity of infections caused by the Beta (Pango: B.1.351) and Gamma (Pango: P.1) VOCs that had a geographically more limited spread. One study found that both Beta and Gamma infections were associated with increased number of hospitalizations and ICU admissions but not with deaths [864]. Another analysis estimated that the odds of death were approximately 1.5-fold higher in infections with the Beta variant compared with the Alpha variant [865]. The Lambda VOC (Pango: C37) fueled a particularly deadly epidemic wave in several countries of South America [873]; however, it is unclear whether or to what extent an increased risk of severe outcome with this VOC might have contributed to the high population-level mortality.

The Delta VOC (Pango: B.1.617.2; dominant lineage worldwide between July and December 2021) seems to have had further increased virulence compared to the first group of successful VOCs (Alpha, Beta and Gamma) that all shared the N501Y mutation in the spike protein. In a cohort of 212,326 patients, individuals infected with the Delta variant (identified by high-probability inference) had an increased chance of hospitalization, admission to ICU, or death, compared with N501Y-positive patients [863]. The probability of hospitalization after a positive test was higher for Delta compared to Alpha infections in two other cohorts [866,867].

After the Delta wave, several lineages of the Omicron VOC (BA.1, BA.2, BA.4, BA.5) were responsible for the next global waves of the pandemic. Analyses of the first Omicron wave (BA.1) reported significantly reduced risk of severe disease compared with the previously circulating Delta variant. Initial results suggested that after Omicron infection the risk of hospital admission decreased by 50–75% and mortality by 80–90% [868,869]. However, it has been pointed out that part of the observed reduction in the severity of COVID-19 can be attributed to the increased ability of the Omicron variants to infect individuals with preexisting immunity, which provides partial protection against severe COVID-19 and death [874]. Analyses taking into account documented antigen exposure and under-ascertainment of prior infections reported a reduction of approximately 30% in the probability of hospitalization, compared with the preceding Delta variant [675,870]. Omicron’s reduced ability to replicate effectively in TMPRSS2 expressing cells such as alveolar tissue (which is the main anatomical site of COVID-19 pathogenesis) and its effective entry into cells of the upper respiratory tract [875,876] (mainly through the cathepsin-L pathway) might explain this reduction in disease severity in immunologically naïve individuals. However, a chimeric recombinant SARS-CoV-2 encoding the S gene of Omicron in the backbone of an ancestral SARS-CoV-2 isolate proved to be highly lethal (similarly to the basal lineage of the virus) in K18-hACE2 mice that experience only mild symptoms when infected with Omicron [877]. This observation suggests that the reduced pathogenicity of Omicron might depend also on changes outside of the spike protein.

Importantly, the population-level impact of Omicron has been shaped by both its reduced per capita risk of mortality and severe disease, and by its increased transmissibility [878], particularly among those with pre-existing immunity, which resulted in higher total case counts. Most studies so far indicate that both BA.2 [872,879,880], BA.4 and BA.5 infected patients [674,881,882,883] show similar severity of COVID-19 compared to BA.1 infections.

The evolution of virulence is hard to predict for the future variants of the virus. Selection does not act directly on the virulence of SARS-CoV-2 (hospitalization and death usually occur after the main transmission period), and its direction most likely depends on how the virus can increase its transmissibility and escape host immune responses [884].

Finally, some attempts have also been made to correlate the severity of infection with individual mutations in the viral genome [885,886,887]. However, the effect of individual mutations is hard to estimate when most comparisons can only be based on observing competing VOCs that differ in multiple mutations that may also involve epistatic effects. We conclude that viral genetic variation can have a strong effect on the risk of severe COVID-19 outcomes, but this cannot easily be traced to individual allelic variants.

### 3.2. Infecting Dose (Inoculum Size)

Accumulating indirect evidence supports the hypothesis that the size of the viral inoculum might influence the outcome of SARS-CoV-2 infection. First, several dose-titration studies using animal models of SARS-CoV-2 (ferrets, mice or Syrian hamsters) have demonstrated an effect of the infecting viral dose on the severity of subsequent disease symptoms [888,889,890].

Second, several observations are compatible with a link between impaired transmission (lower infecting dose) and lower frequency of severe disease in humans. In particular, widespread use of masking (which is likely to reduce the infecting dose) appears to be associated not only with reduced transmission, but also with a reduced severity or frequency of symptoms among the remaining cases [891,892]. There are also several case studies in closely monitored settings that found an increase in the rate of asymptomatic infections following the introduction of masking, for example at a seafood processing facility and at a chicken plant [891]. Finally, in addition to masking, there are further documented cases where highly similar groups developed divergent clinical forms of COVID-19 potentially due to differences in the setting of exposure. In a study conducted in Spain, among three different clusters of infection the outcome of the disease was the mildest where individuals lived in a large house, less benign where they stayed in an apartment flat, and most severe in the case of attendees of a pre-lockdown meeting in a small conference hall [893]. A Swiss study compared outbreaks in a military setting before and after the introduction of social distancing and stringent hygiene measures [894]. In two of the three groups of young, predominantly male soldiers, the first outbreak occurred before the introduction of preventive measures, and in these groups, 30% of cases resulted in a symptomatic infection. In contrast, the third group experienced their first outbreak after the measures had been implemented, and all infections were asymptomatic.

One hypothesis for the possible causal link between higher infecting dose and more severe disease posits that a larger viral inoculum might overwhelm and evade the primary innate immune responses, resulting in the release of high levels of inflammatory mediators [895]. A larger initial dose might also allow the virus to replicate to higher levels before adaptive immune responses are launched. Note that these mechanisms do not depend on the specifics of COVID-19, and indeed, similar dose dependence of symptoms has been documented for human influenza viruses [896,897], SARS-CoV [898], respiratory syncytial virus [899] and for several non-human pathogens (see reviewed in [892,900]), which lends further indirect support to the “SARS-CoV-2 inoculum hypothesis” [895].

The route of transmission might also influence the clinical outcome of COVID-19. Multiple studies using animal models of COVID-19 indicate that airborne and especially aerosol transmission might result in a disease with an earlier onset and higher severity compared to infections acquired by fomite, oral or gastrointestinal exposures [888,901,902,903].

We conclude that current support for the dose response of COVID-19 severity arises from indirect evidence, and the magnitude of the effect is unclear. An ongoing human dose finding infection study [904] might soon yield the first direct estimation of the effect size.

## 4. Environmental Factors

### 4.1. Socioeconomic Factors

Socioeconomic status (SES) has a strong impact on general health and life expectancy [905,906], and could therefore be expected to affect the risk of severe COVID-19 as well. There are an estimated 435 million people in low-income countries (mostly in Sub-Saharan Africa, East and South Asia) who are at high risk from COVID-19 due to their lack of access to health care and safe drinking water, exposure to household air pollution, undernutrition, and other factors associated with low SES [907]. In addition, regional and individual-level studies reported a disproportionately high share of ethnic minorities among both COVID-19 cases and deaths in high-income countries [105,908,909,910,911], which also hints at the importance of multidimensional poverty in COVID-19 morbidity and mortality. Such effects may arise from differences in both the risk of acquiring the infection, and the risk of severe outcomes when infected.

A large body of accumulating evidence supports the idea that low SES is an independent risk factor of severe COVID-19. Multiple indicators related to housing, poverty, nutrition, health care, education and belonging to an ethnic minority have been associated with the outcome of the disease [104,911], and although not all studies confirmed the effects [912,913,914,915] (Table 7), the associations were typically stronger in the larger studies (death in the lowest income quantile compared to the highest OR = 1.95 (1.56–2.43) [202], OR = 1.79 (1.68–1.80) [281]). Although low SES is connected to several other COVID-19 severity risk factors (see Section 5.1. *Interactions Between Risk Factors of Severe COVID-19*), its independent effect on COVID-19 outcomes indicates that not all aspects of SES relevant for the course of the infection are captured in the known risk factors associated with it.

We note that socioeconomic factors are likely to contribute to the effect of ethnicity/ancestry on COVID-19, due to statistical associations between the two factors. Most meta-analyses (relying mostly on data from the UK and the US) have failed to show an independent effect of ethnicity/ancestry on mortality among hospitalized cases [108,926,927] (except [104]), implying that the higher COVID-19 fatality rates observed among some ethnic minorities might be explained mainly by the higher prevalence of certain comorbidities [928,929,930,931,932] and low SES [933] in these groups. For this reason, and in the absence of a strong case for genetic differences influencing COVID-19 severity that could be linked to ancestry (see Section 2.2. *Human Genetic Variation*), we do not discuss ethnicity/ancestry as an independent host factor of severe COVID-19.

### 4.2. Air Pollution

Air pollution has been a public health issue since the industrial revolution [934]. One and a half centuries of chemical manufacturing has resulted in an increased concentration of pollutants in the air, mainly in the form of SO_2_, NO_2_, NH_3_, CO, O_3_, volatile organic compounds and particulate matter (PM).

The association of the environmental concentration of these pollutants with COVID-19 severity has been extensively investigated (summarized in Table 8), with some, but not all, studies demonstrating statistically significant associations.

While the results of the direct association studies have been mixed, several lines of indirect evidence support that air pollution may have some detrimental effect on COVID-19 pathogenesis. Several components of particulate air pollution facilitate the formation of reactive oxygen signals, which causes inflammation in the lungs [950]. Inflammation caused by PM, and COVID-19 pathogenesis share multiple signaling pathways (TLR, NLR, Nrf2, NF-κB, TNF, IL-1, IL-17, and JAK-STAT) [951,952], which further reinforces this connection. The enhancement of pulmonary epithelial permeability, suppression of mucociliary clearance, interference with antimicrobial proteins, induction of antibacterial instead of antiviral innate immune responses, induced mitochondrial damage and apoptosis, inhibition of IFN production and overexpression of inflammatory metabolites have all been proposed as potential mechanisms [891,953,954]. PM2.5-mediated upregulation of ACE2 in the lungs has also been suggested and supported by an animal study [955] and a human cell culture experiment [956], but a bioinformatic analysis of transcriptomic data related to COVID-19 lung biopsy, SARS-CoV-2 infection in epithelial cells and PM exposure failed to find significant ACE2 upregulation in human infections [952].

We conclude that the link between air pollution and COVID-19 clinical outcome has relatively weak direct evidence, but may affect some subpopulations (e.g., with certain comorbidities) more strongly.

## 5. Interactions between Effects

### 5.1. Interactions between Risk Factors of Severe COVID-19

**Figure 1:**
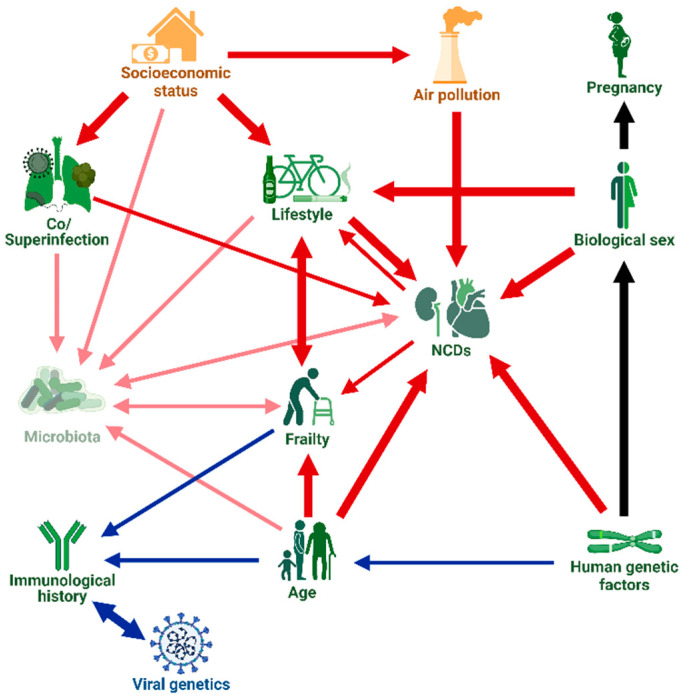
The network of interactions between identified and proposed risk factors of severe COVID-19. Host (green), viral (blue) and environmental (yellow) factors are connected by arrows indicating three types of interactions between the factors: some factors might modulate the effect of other factors (blue arrows), e.g., advanced age shortens the duration of effective adaptive immune responses; some interactions may affect the occurrence of factors (red arrows), e.g., lifestyle choices influence the likelihood of certain comorbidities; finally, some factors act as the major determinant of another (black arrows), e.g., biological sex determines the possibility of pregnancy, and sex itself is genetically determined in humans. The width of the arrows indicates the perceived magnitude of these effects. The pictogram of microbiota alterations is faded out to illustrate the uncertainty of its causal role in the development of severe COVID-19. The figure was created with BioRender.com.

Risk factors of severe COVID-19 do not act in isolation but may influence both the impact and the occurrence of each other in multiple ways (Figure 1). The effect of low socioeconomic status is likely to be mediated, at least in part, through known risk factors that tend to be related to poverty. Low SES is associated with increased chronic and respiratory infection burden both among adults [957,958] and children [959,960], predisposing these disadvantaged individuals to NCDs. Furthermore, multidimensional poverty has a deteriorating effect on lifestyle (nutrition [961,962,963], smoking [964,965,966]), microbiota diversity and composition [967,968,969], and on the development of several comorbidities (cardiovascular [970], pulmonary [971], renal [972], metabolic [973,974] diseases and cancer [975]). Poor housing conditions can be associated with increased indoor air pollution [976,977], which increases the prevalence and severity of both chronic respiratory conditions [978,979] and respiratory infections [978,980,981]. Overcrowding, low-SES neighborhood characteristics and financial instability also escalate chronic stress levels [957,982,983,984], which is considered to be a main contributor to premature mortality in socioeconomically disadvantaged individuals [985,986,987], and may also affect COVID-19 outcomes.

Lifestyle choices influence the development of non-communicable diseases (CVD [988,989], obesity [990], DM [991], CKD [992] and CLD [993]) independently from socioeconomic status, and they also affect the deterioration of physical functions (frailty) [994,995] and the composition and diversity of the gastrointestinal microbiota [996]. A few studies also examined the modulating effect of physical activity and dietary choices on COVID-19-related microbial alteration [594,997].

Air pollution, and long-term PM exposure in particular, have been linked to decreased lung function [998,999] and a number of comorbidities, such as asthma [1000], COPD [1001], cardiovascular disease [1002], lung cancer [1003], and type 2 diabetes mellitus [1004]. Subpopulations with respiratory diseases show a stronger association between air pollution and COVID-19 severity [940,941,943], which further supports the interaction of these two factors. In turn, chronic lung diseases can limit the capacity of patients for physical work, and affect lifestyle [1005,1006].

Certain chronic coinfections predispose to NCDs (e.g., HIV can facilitate the development of CVD, CKD, CLD, cancer and secondary immunodeficiency [1007], while hepatitis viruses promote liver cirrhosis and cancer [1008]). Furthermore, some parasite infections cause alterations in microbiota diversity [1009,1010], which is tightly connected with the regulation of inflammatory responses [1011,1012].

The gastrointestinal microbiota play an important role in several chronic diseases that are associated with the outcome of COVID-19, such as type 2 DM [1013,1014], obesity [1015,1016], CHD [1017,1018], hypertension [1019,1020], lung diseases (allergies, asthma, COPD) [1021,1022], CLD (liver cirrhosis [1023] and NAFLD [1024]), CKD [1025], neurodegenerative diseases [1026] and depression [1027]. Similarly to the effect of age, NCD- and frailty-related dysbiosis in the GIT [1028,1029,1030] correlates with increased sterile inflammation and inflamm-aging [1031,1032], low butyrate production [1033,1034], impaired barrier function [1035] and increased lipopolysaccharide (LPS) levels in the blood [1036].

Frailty syndrome is characterized by an increased level of sterile systemic inflammation and diminished immune responses, and it is closely related both to age and to age-related diseases [1037]. The deterioration in the physical condition of frail individuals makes it harder to maintain physical activity, possibly accelerating the decline in physical health [1038]. A large proportion of frail individuals have one or more comorbidities, which indicates an overlap between these two risk factors [1039,1040]. As an important example, metabolic inflammation [1039], due to the accumulation of adipose tissue, contributes to the observed inflammatory phenotype in frail individuals [1031]. Although frail individuals have functional adaptive immune responses after SARS-CoV-2 infection [1041] and vaccination [1042,1043], faster waning of IgG levels [1044,1045] and immune-senescent memory T cell functions [1046] indicates that immune memory might be less durable compared to in non-frail individuals.

Age and inflamm-aging is associated with comorbidities, such as obesity, atherosclerosis, rheumatoid arthritis, diabetes, and neurodegeneration [1047,1048]. Reduced ability to recognize cellular damage and the build-up of senescent cells during aging also contribute to the observed inflammatory phenotype in frail individuals [1031]. Aging is also likely to modulate the protective effect of both specific immunity from previous episodes of COVID-19 [677], and cross-reactive immunity from other infections [1049], and is associated with shifts in the upper respiratory tract (URT) [604] and GI microbiota [1050]. Compositional changes and the low diversity of the microbiota in advanced age are associated with a weakened intestinal barrier [1051], elevated levels of bacterial products (such as LPS) in the blood [1052] and heightened inflammation (inflamm-aging) [1053,1054]. In turn, preservation of the microbiota in elderly individuals is correlated with slower immunosenescence [1055]. One study found that age modifies the correlation between microbiota changes and COVID-19 symptom severity concluding that dysbiosis might be an important mediating mechanism between age and COVID-19 severity [604].

Women tend to attribute higher priority to physical health [1056,1057], be more prone to adapt a healthy diet [1058], adhere to hygiene habits [1059], seek professional care [1060] and comply to its recommendations [1061] compared with men [1062,1063,1064]. These behavioral and lifestyle factors contribute to the higher prevalence of chronic diseases, such as COPD [1065], obesity [1066], DM [1067], hypertension [1068] and CVD [1069] among men in high-income countries. However, sex differences in the severity of COVID-19 cannot be explained fully with differences in the prevalence of lifestyle-associated NCDs and health behaviors [1070], indicating further, independent effects of this factor.

Human genetic polymorphisms influence healthy aging and longevity [1071,1072], and predisposition for certain comorbidities, such as type I [1073,1074] and II [1075,1076] diabetes mellitus, obesity [1077,1078] and cardiovascular disease [1079,1080]. Several polymorphisms involved in the SARS-CoV-2-human protein contactome have been associated also with non-communicable diseases (cardiovascular diseases, obesity, schizophrenia) [1081].

Lastly, there is an interaction between the effects of specific immunity from past SARS-CoV-2 infections and viral variation, since the antigenic match between the strain(s) involved in the previous and the current exposures can modulate the protective effect of immunity [1082].

### 5.2. Direct and Indirect Effects of COVID-19 on the Risk Factors of Severe Disease

While the severity of COVID-19 is influenced by the factors discussed in this review paper, this interaction can be bidirectional, as some of the risk factors can themselves be affected either directly by COVID-19, or indirectly by the human interventions aimed at mitigating the impact of the epidemic. COVID-19 can lead to lasting damage to physical health, which can involve some of the risk factors as well. Acute organ injury caused by COVID-19 can cause long-term cardiovascular [1083,1084], pulmonary [1085], metabolic [1086], renal [1086] and neurological [1087] damage, potentially contributing to chronic conditions that can themselves be risk factors for severe COVID-19 in subsequent infections. COVID-19 can lead to the exacerbation of asthma [1088] and neurodegenerative disorders (both Parkinson’s and Alzheimer’s disease) [1088], and increase the risk of developing mental disorders [1088,1089], diabetes [1090,1091], CKD [1092], hypertension [1093] and CVD [1094]. COVID-19 also disturbs metabolic homeostasis, and can result in a low intake of calories and greatly reduced physical activity during hospitalization, which can exacerbate frailty and biological aging [586]. Indeed, in small cohorts, the incidence of frailty and the number of disabilities increased after COVID-19 hospitalization and critical care [1095,1096,1097].

In addition to the direct pathology of COVID-19, long-term stringent lockdown measures might also contribute to the increased prevalence of some COVID-19 risk factors. During lockdown periods, physical activity levels tend to decrease substantially [1098,1099], while the sales [1100] and consumption [1101] of alcoholic beverages tend to increase in several populations (e.g., older people, more depressed individuals, and essential workers) [1102], indicating that drinking might be a coping mechanism for some during social isolation [1103]. Finally, the economic consequences of the pandemic have negatively impacted the financial situation of hundreds of millions of people worldwide [1104,1105]. Those who had already been at the edge of poverty [1106,1107,1108,1109] or were not able to work from home [1110,1111] had been affected most strongly. Severely affected households experienced increasing food insecurity [1105,1108,1112] and deteriorating mental [1113,1114,1115,1116] and cardiovascular health [1117]. The effects of the pandemic on SES have thus created a positive feedback loop by putting millions of people into a socioeconomic position that makes them more susceptible to both SARS-CoV-2 infection and severe COVID-19.

## 6. Discussion and Conclusions

In this review, we aimed to provide a comprehensive overview of all ‘inherent’ risk factors of severe COVID-19, omitting only the effect of medical interventions (vaccination and therapy). This broad scope of the review entails some inevitable limitations: while we attempted to identify the most relevant studies for each factor, it was not feasible to conduct a systematic review of all factors. Furthermore, the definitions of ‘mild’ and ‘severe’ COVID-19, the markers used to quantify disease severity, and the potential confounding factors included in the analyses differed widely between the studies, which makes a systematic comparison and summary of this immensely broad field practically unfeasible. The study populations might also have differed in hidden background variables, and the sheer number of the potential contributing factors makes the unbiased estimation of the effect of individual factors very hard. To point the reader to the (in our subjective assessment) most reliable sources of more detailed information, we provide a selection of the largest and (in terms of the cofactors considered) most comprehensive cohort studies and meta-analyses that investigated multiple risk factors of severe COVID-19 in Supplementary Table S1.

Possibly due to the difficulties outlined above, the importance of several factors in COVID-19 remains controversial, with conflicting results in the published literature. Moreover, it is often hard to establish causality and to elucidate the causative mechanisms behind the associations identified. For each factor, we outlined the proposed (in some cases, largely hypothetical) mechanisms of action, and we discussed potential interactions (involving indirectly mediated effects) between the factors.

Based on our broad survey of host, viral and environmental factors implicated in the risk of severe COVID-19, we conclude that both in terms of the strength of the evidence and in the number of infections affected, the age and sex of the patient, the common comorbidities grouped as ‘metabolic syndrome’ (diabetes, hypertension, obesity), and the inherent propensity of the viral variant (VOC) to cause severe disease stand out as the most important factors for the first encounter with the virus, while subsequent re-infections are also strongly affected by SARS-CoV-2-specific adaptive immunity. Influenced by variation in these factors, the risk of individuals varies across a very broad spectrum (age alone spanned approximately four orders of magnitude in the risk of mortality in the first wave of the epidemic [9]). Awareness of these factors can help decisions in the clinic (whom to treat, how to allocate resources), and also public health decisions at the population level. While prevalent immunity from previous infections and vaccinations (and the emergence of the Omicron variants) has substantially reduced the overall risk of severe outcomes compared with the first waves of the pandemic, the risk factors identified in previous analyses are likely to continue to shape relative risks in the future.

## Figures and Tables

**Table 1:** Genes with rare or common variants that have highly supported association with COVID-19 severity.

Functional Category	Gene or Genetic Region	Severity	Function Related to COVID-19 Pathogenesis *	Sources ^#^
Direct interaction with SARS-CoV-2	ACE2 ^†^	increased/decreased	facilitation of SARS-CoV-2 cell entry, regulation of cardiovascular and renal function	[63]
	TMPRSS2 ^†^	increased/decreased	facilitation of SARS-CoV-2 cell entry	[64,65,66]
Respiratory surface barrier	MUC1	increased	formation of respiratory mucosal barrier	[67,68]
	MUC5B ^†^	decreased	the major gel-forming mucin in mucus	[67,69]
	LZTFL1 ^†^/(SLC6A20)	increased	regulation of protein trafficking to the ciliary membrane/(proline transportation in the kidney and small intestine)	[67,68,70,71,72]
	NAPSA/KCNC3	increased	may be important in the processing of pulmonary surfactant protein B/mediates the voltage-dependent potassium ion permeability of excitable membranes	[68]
Immunity	HLA region ^†^	increased/decreased	recognition and presentation of tolerogen and immunogen protein epitopes	[64,67,68,71,73]
	SFTPD	increased	innate immune protein in the lungs	[67]
	OAS1 ^†^/(OAS3)	increased	innate cellular antiviral responses/(viral infection resistance)	[67,68,71,74,75]
	DPP9	increased	role in MHC-I peptide presentation	[67,68,70,71]
	TYK2	increased	Janus-kinase in cytokine signalization pathways	[67,68,71]
	IFNAR2 ^†^	increased	interferon receptor formation for IFN-alpha and -beta	[67,68,70,71]
	TLR7 ^†^	increased	recognition of single-stranded RNA viruses in the endosomal system	[67,76]
	DOCK2 ^†^	increased	remodeling of the actin cytoskeleton required for migration in response to chemokine signaling in peripheral blood leukocytes	[77]
Regulation of blood pressure	TAC4/KAT7	increased	receptor activation → regulation of blood pressure, the immune system, and endocrine gland secretion/part of a complex with acetyltransferase activity	[68,71]
	ACE1 ^†^	increased/decreased	regulation of blood pressure and electrolyte levels	[64,65,78]
Other	FOXP4	increased	regulation of gene transcription on the cell and tissue levels	[67,68,71]
	ELF5 ^†^	increased	epithelium-specific gene regulation and differentiation of keratinocytes	[67,79]
	KANSL1/WNT3	increased	role in histone acetylation → cell proliferation, mitosis/developmental regulation	[71]
	ABO ^†^	increased/decreased	blood group determination	[68,80]
	ApoE ^†^	increased	component of chylomicron, catabolism of triglycerides	[64,81]
	FBRLS1	increased	neurological and non-neurological functions	[67]

* We collected information about the physiological role of the listed genes from the Gene library of the NCBI database [82]. ^#^ Since this field is intensively researched, in some cases, we cite only systematic and narrative reviews on the association of an allele with COVID-19 severity. ^†^ The effect of these genes has been demonstrated by targeted analyses, in addition to high-throughput screening.

**Table 2:** Large clinical studies and systematic reviews demonstrating the association of male sex with increased COVID-19 severity.

Study Design	Outcome	N of Cases	Covariates	Effect	Source
Retrospective cohort	severe disease	174,568	age, race, ethnicity, insurance status, weight, BMI	aOR = 1.60 (1.51–1.69)	[120]
Retrospective cohort	mortality	116,539	age, presence of comorbidities	aOR = 1.42 (1.38–1.47)	[121]
Prospective cohort study	hospitalization	16,475	age, comorbidities, education level, income, work status	aHR = 1.63 (1.57–1.68)	[115]
Cross-sectional	ICU admission	14,992	age, race, ethnicity, marital status, insurance type, median income, BMI, smoking and 17 comorbidities	aOR = 1.39 (1.23–1.59)	[118]
Systematic review	critical outcome	43,248	-	pRR = 1.26 (1.17–1.36)	[122]
Systematic review	mortality	423,117	most studies collected information on covariates	pHR = 1.24 (1.07–1.41)	[123]
Systematic review	severe disease	21,060	39 out of 41 studies adjusted for at least 3 covariates	pOR = 1.51 (1.33–1.71)	[124]
Systematic review	severe disease	~440,000	most studies collected information on covariates	pOR = 2.05 (1.39–3.04)	[11]

Abbreviations: OR—odds ratio; HR—hazard ratio; RR—risk ratio; aOR and aHR—odds and hazard ratio adjusted for covariates; pOR, pHR, pRR—odds, hazard and risk ratios estimated in pooled analysis.

**Table 3:** Prevalent comorbidities with highly supported association with the risk of in-hospital COVID-19 mortality. The sources shown are either meta-analyses that found a significant summary effect, or large clinical studies.

Category	Comorbidity	Covariates	Effect	Source
respiratory	chronic obstructive lung disease (COPD)	age, sex, income, urbanization, LTC residency, comorbidities	HR = 1.19 (1.12–1.26)	[201]
		age, sex, socioeconomic status, ethnicity, health care service, comorbidities	OR = 1.35 (1.27–1.42)	[202]
		Variable *	OR = 1.25 (1.08–1.34)	[203]
cardiovascular	hypertension	age, sex, income, urbanization, LTC residency, comorbidities	HR = 1.16 (1.07–1.26)	[201]
		age, sex, socioeconomic status, ethnicity, health care service, comorbidities	OR = 1.39 (1.35–1.44)	[202]
		variable *	RR = 1.42 (1.30–1.54)	[204]
	cardiovascular disease (CVD)	age, sex, income, urbanization, LTC residency, comorbidities	HR = 1.22 (1.15–1.30)	[201]
		age, sex, socioeconomic status, ethnicity, health care service, comorbidities	OR = 1.06 (1.02–1.12)	[202]
		variable *	OR = 3.11 (2.55–3.79)	[205]
renal	chronic kidney disease (CKD)	age, sex, income, urbanization, LTC residency, comorbidities	HR = 1.45 (1.34–1.57)	[201]
		age, sex, socioeconomic status, ethnicity, health care service, comorbidities	OR = 2.40 (2.30–2.51)	[202]
		variable *	OR = 5.81 (3.78–8.94)	[206]
metabolic	diabetes (DM)	age, sex, income, urbanization, long-term care (LTC) residency, comorbidities	HR = 1.19 (1.12–1.26)	[201]
		age, sex, socioeconomic status, ethnicity, health care service, comorbidities	OR = 1.82 (1.76–1.88)	[202]
		variable *	RR = 1.54 (1.44–1.64)	[204]
	obesity	age, sex, socioeconomic status, ethnicity, health care service, comorbidities	OR = 1.68 (1.62–1.73)	[202]
		variable *	RR = 1.45 (1.31–1.61)	[204]
		variable *	OR = 1.61 (1.29–2.01)	[207]
other	cancer	age, sex, income, urbanization, LTC residency, comorbidities	HR = 1.17 (1.09–1.27)	[201]
		variable *	OR = 1.71 (1.539–1.905)	[208]
		variable *	RR = 1.44 (1.19–1.76)	[209]

* The individual studies included in the meta-analyses varied in the covariates considered. Abbreviations: OR—odds ratio; HR—hazard ratio; RR—risk ratio.

**Table 4:** Comorbidities with weakly supported or controversial association with COVID-19 severity.

Comorbidity		Studies Reporting Correlation with COVID-19 Outcome	Studies Reporting Lack of Correlation with COVID-19 Outcome
asthma		death [247]	Severity * [269], hospitalization * [239,270], ICU * [239], death * [202,208,239,271,272,273,274,275], severity [276], hospitalization [203,277,278], ICU [203,277,278], MV [274,278], death [13,201,203,270,277,278,279,280,281]
interstitial lung disease (ILD)		severity [231], ICU [232], death [232,233,234]	hospitalization [232], MV [232,233]
coronary heart disease (CHD)		severity [124,282], ICU [283], death [283,284]	death [285,286]
chronic liver disease (CLD)		hospitalization [287,288], severity [120,124,289,290], ICU [291], MV [291], death [208,280,281,287,288,292]	severity [293,294], death [201,280,291,295]
	liver cirrhosis	severity [296], death [287,296,297,298,299]	severity [296,300], death [300]
	metabolic associated/non-alcoholic fatty liver disease (MAFLD/NAFLD)	severity [301,302,303,304,305,306,307,308], ICU [291,308], MV [291], death [309]	ICU [307], death [291,308,309,310]
	alcohol-related liver disease (ALD)	death [299,310]	severity [299]
immune-mediated inflammatory disease (IMID)/autoimmune disease		hospitalization [311], severity [312]	severity [313,314], MV [315], death [313,314,315,316,317]
	rheumatoid arthritis (RA)	hospitalization [288], severity [201,290], death [280,281,288]	severity [318,319], ICU [319], MV [319], death [201,318,319]
immunosuppression		hospitalization [320,321], severity [322], ICU [323], death [13,280,281,321,324,325,326]	hospitalization [320,321], severity [327], MV [315], death [315,321,323,326]
	organ transplant	hospitalization [328], severity [269,329], ICU [323,330], death [201,280,281,329,331]	severity [332,333], death [323,330,334,335,336,337]
asplenia		death [281]	death [280,281,338]
cognitive disorder		severity [339], death [339]	-
	dementia	hospitalization [340], severity [120,341], death [201,280,286,339,340,341,342,343,344,345,346,347,348]	severity [339], ICU [349]
	Alzheimer’s disease (AD)	severity [350,351], death [342,343,344,350,352]	MV [351]
cerebrovascular disease (CeVD)		severity [124,269,290,293,353,354,355,356,357], ICU [355,358], MV [358], death [354,355,356,358]	-
	stroke	severity [359], death [280,359,360,361]	severity [339], death [339]
epilepsy		severity [362], death [339]	severity [339], death [362]
obstructive sleep apnea (OSA)		hospitalization [346], severity [290,363,364,365], ICU [363], MV [363], death [363,366]	MV [367], death [367]
Parkinson’s disease (PD)		severity [350]	hospitalization [368], death [342,350,369]
mood disorders		hospitalization [370], severity [339], death [339,370,371,372]	severity [370]
	bipolar disorder	hospitalization [288,373], severity [374], death [288,344,373,374,375]	-
	major depressive disorder/depression	hospitalization [288,370,376,377], severity [365,374], death [286,288,344,370,374,376]	hospitalization [378], severity [339,370], death [371]
psychotic disorders		hospitalization [379,380], death [339,371,376,380]	hospitalization [376], severity [339], death [344]
	schizophrenia	severity [374], death [339,374,381,382,383]	hospitalization [378], severity [339]
stress-related disorder		-	hospitalization [376], death [339,376]
substance use disorders		hospitalization [376,379,384,385], MV [384], death [371,384]	ICU [385], death [344,376,385]
attention deficit hyperactivity disorder (ADHD)		severity [339,378], death [378]	death [339]

* Negative correlation between the chronic comorbidity and severe COVID-19 outcome. Abbreviations: ICU—intensive care unit; MV—mechanical ventilation.

**Table 5:** Co- and superinfections tested for associations with COVID-19 severity.

Group	Species	Potential Factors Facilitating Co/Superinfection	Effect	Potential Mechanisms *
Bacteria	Pneumonia causing bacteria ^#^	dysbiosis [426], disrupted epithelial barrier [427], hyperactive immune response [427], NET degradation [428], mechanical ventilation [429]	increased severity [430,431,432,433]not associated [434,435,436]	↑ exacerbation of inflammation [437,438] and pneumonia [439,440,441], ↑ reduced T cell, B cell and mucosal IgA responses [437]
	*Mycobacterium tuberculosis* ^†^	increased attachment and colonization due to weakened immunity [442]	increased severity [443,444,445]not associated [446,447]	↑ exacerbation of inflammation [442,448], ↑ upregulated IFN responses [449,450], ↑ depletion of immune cells targeting MT [442,448,451], ↑ interference with SARS-CoV-2-specific immunity [452], ↓ heterologous immunity [446], ↓ lower risk of immune-mediated damage [448]
Viruses	HBV ^#^	increased HBV reactivation in immunosuppressed patients [453], but not in general [454]	increased severity [455]not associated [443,456]	↑ higher risk of liver injury [455,457,458,459], ↓ suppression of overactive immune responses [460]
	HCV ^†^	both utilize structurally similar ion channels [461]	not associated [462]	↑ heightened inflammation [463,464], ↑ vascular endothelial dysfunction [465], ↑ extrahepatic damage [466], ↑ liver cirrhosis [467]
	HIV ^†^	uncontrolled infection [468]	increased severity ^‡^ [445,469,470,471,472,473,474,475]not associated [443]	↑ uncontrolled infection [468,471,476,477,478] with reduced B cell functions [479,480,481,482,483], lymphopenia [451,471,476,478,484], chronic inflammation [485,486] and comorbidities [477,487]
	influenza viruses ^†^	interferon-induced overexpression of ACE2 [488,489]	increased severity [490,491]not associated [492]	↑ increased inflammation [493,494,495,496], ↓ viral interference through antibodies [492,497,498,499], or interferon effects [500,501]
	HRV ^†^	a HRV serotype overexpresses ACE2 and TMPRSS2 on epithelial cells [502]	not associated [503,504]	↓ induced epithelial IFN responses block SARS-CoV-2 replication [505,506]
Fungi	*Aspergillus* spp. ^#^	dysregulated immune system (corticosteroids, lymphopenia) [507,508]	high reported CFR [509,510]	↑ exacerbation of pneumonia (IL-6 [511,512], IL-10 [513,514])
	*Candida* spp. ^#^	dysregulated immune system (corticosteroids, lymphopenia) [507,508], mechanical ventilation [508], antibiotic use [508]	increased severity [431]	↑ exacerbation of pneumonia [515] (IL-6 [516,517])
Parasites	Helminths ^†^	altered mucus secretion [518]	reduced severity [519]	↓ induced Th2 responses [520], ↓ attenuated sepsis [520], ↓ increased microbiota diversity [521,522], ↑ inability to produce early immune responses [520], ↑ nutritional and metabolic problems [523]
	*Entamoeba and Giardia* spp.	-	reduced severity [519]	↓ induced Th2 responses [522], ↓ increased diversity of microbiota or ↑ dysbiosis [522]
	*Plasmodium* spp.	-	increased severity [524]	↑ T cell exhaustion [525] ↑ fewer atypical memory B cells [526], ↓ cross-reactivity [527]
	*Trypanosoma* spp.	-	not associated [528,529]	↓chronic but regulated inflammation [528]

Abbreviations: HBV—hepatitis B virus, HCV—hepatitis C virus, HIV—human immunodeficiency virus, HRV—human rhinovirus, and CFR—case fatality ratio. * Mechanisms implicated in increased (↑) or decreased (↓) severity are indicated by arrow symbols (even in those cases where significant effect had not been reported). ^#^ In the case of these pathogens, SARS-CoV-2 infection facilitates co/superinfection by the indicated pathogen. ^†^ These species/groups facilitate co/superinfection by SARS-CoV-2. ^‡^ Where available, poorer HIV clinical status (lower CD4 count, uncontrolled vs. controlled HIV viremia) was associated with a stronger effect on COVID-19, that is a more increased risk of severe COVID-19 outcomes [445,471,473].

**Table 6:** Clinical studies investigating the impact of SARS-CoV-2 VOCs on COVID-19 disease severity compared to non-VOC or previous VOC.

VOC	Reference	Covariates	Outcome	n	Effect (95% CI)	Source
Alpha	non-VOC	age, sex, ethnicity, index of multiple deprivation, lower tier local authority region, test date	death	54,906	aHR = 1.64 (1.32–2.04)	[861]
Alpha	non-VOC	age, sex, ethnicity, deprivation, residence in a care home, the local authority of residence, test date	death	1,146,534	aHR = 1.55 (1.39–1.72)	[862]
Probable Alpha (N501Y+)	non-VOC	age, sex, time, vaccination status, comorbidities, and pregnancy status	hospitalization	162,854	aOR = 1.52 (1.42–1.63)	[863]
			ICU		aOR = 1.89 (1.67–2.17)	
			death		aOR = 1.51 (1.30–1.78)	
Beta	non-VOC	age, sex, week of reporting, country	hospitalization	436	aOR = 3.6 (2.1–6.2)	[864]
			ICU		aOR = 3.3 (1.9–5.7)	
			death		aOR = 1.1 (0.4–3.4)	
Beta	Alpha	age, sex, diagnosis date	severe	9182	aOR = 1.24 (1.11–1.39)	[865]
			critical		aOR = 1.49 (1.13–1.97)	
			death		aOR = 1.57 (1.03–2.43)	
Gamma	non-VOC	age, sex, week of reporting, country	hospitalization	352	aOR = 4.2 (2.1–8.4)	[864]
			ICU		aOR = 2.2 (1.8–2.9)	
			death		aOR = 0.6 (0.3–1.0)	
Delta	non-VOC	age, sex, time, vaccination status, comorbidities, and pregnancy status	hospitalization	5945	aOR = 2.08 (1.78–2.40)	[863]
			ICU		aOR = 3.35 (2.60–4.31)	
			death		aOR = 2.33 (1.54–3.31)	
Delta	Alpha	age, sex, relative socioeconomic deprivation, ethnicity	hospitalization	8682	aHR = 2.26 (1.32–3.89)	[866]
			hospitalization/emergency care		aHR = 1.45 (1.08–1.95)	
Delta	Alpha	age, sex, deprivation, test date, comorbidities	hospitalization	9996	aHR = 1.85 (1.39–2.47)	[867]
Omicron (BA.1)	Delta	sex, age, previous infection, vaccination status, Charlson comorbidity index	hospitalization	6581	aHR = 0.25 (0.15–0.43)	[868]
			death		aHR = 0.14 (0.0011–1.12)	
Omicron (BA.1)	Delta	age, sex, race/ethnicity, and neighborhood-level median household income, smoking, body mass index, Charlson comorbidity index, health care utilization	hospitalization	52,297	aHR = 0.48 (0.36–0.64)	[869]
			death		aHR = 0.09 (0.01–0.75)	
Omicron (BA.1)	Delta	age, sex, comorbidities, geography, vaccination, prior infection (corrected for under-ascertainment)	hospitalization/death	5144 *	aHR = 0.72	[870]
Omicron (BA.1)	Delta	reinfection (corrected for under-ascertainment of prior infections), vaccination status, 10-year age-band, sex, ethnicity, NHS region, specimen date	hospitalization	55,583	aHR = 0.65	[675]
Omicron (BA.1)	Omicron (BA.2)	age, comorbidities, vaccination, ethnicity and race, sex, previous infection status	hospitalization	1720	aOR = 2.71 (2.42–3.02)	[871]
			ICU	232	aOR = 3.06 (2.28–4.10)	
			MV	272	aOR = 3.55 (2.61–4.84)	
			death	203	aOR = 2.20 (1.56–3.11)	
Omicron (BA.2)	Omicron (BA.1)	age, sex, comorbidities, geography, health care sector, and previous SARS-CoV-2 infection	hospitalization	8276	aOR = 0·96 (0·85–1·09)	[872]
Omicron (BA.4/BA.5)	Omicron (BA.1)	age, sex, comorbidities, geography, health care sector	hospitalization	1806	aOR = 1.24 (0.98–1.55)	[674]

* Number of cases during wave 4 in Western Cape Province, South Africa. Abbreviations: OR—odds ratio; HR—hazard ratio; RR—risk ratio, VOC—variant of concern, ICU—intensive care unit, and n—number of VOC cases in a study.

**Table 7:** Socioeconomic variables implicated in the outcome of COVID-19: studies with or without evidence of association.

SES Indicator	Studies Reporting Correlation with COVID-19 Outcome	Studies Reporting Lack of Correlation with COVID-19 Outcome
Poverty	hospitalization [106,109,202], severe disease [916], length of stay [917], ICU [106], MV [917], mortality [109,202,280,281,912,918,919,920,921]	hospitalization [914,915], severe disease [922], ICU [912], MV [202], mortality [106,201,915]
Nutrition	severe disease [819,825], mortality [918]	severe disease [819,916]
Health care	severe disease [913], ICU [912], mortality [109,918]	mortality [912]
Education	mortality [918]	-
Minority status	hospitalization [106,109,914,917,923,924], severe disease [106,912,913,925], ICU [912], mortality [109,923,925]	hospitalization [923], ICU [106], mortality [106,912,915,921,923],
Housing	mortality [918]	severe disease [916]

Abbreviations: ICU—intensive care unit; MV—mechanical ventilation.

**Table 8:** Individual-level studies investigating the association between air pollutant exposure and risk of severe clinical outcomes with COVID-19.

Pollutant	Duration	Studies Reporting Association with COVID-19 Outcome	Studies Reporting Lack of Correlation with COVID-19 Outcome
B(a)P	Short term	mortality [935]	MV [935]
BC	Long term	-	severe disease [936], ICU [937], MV [937], mortality [937]
PM2.5	Short term	mortality [935]	MV [935], mortality [938,939]
	Long term	hospitalization [940,941,942], severe disease [936,943], ICU [937], mortality [937,939]	hospitalization [940,942,944], ICU [942], MV [937], mortality [942,945,946]
PM10	Short term	pneumonia [947]	MV [935], mortality [935]
	Long term	severe disease [948], mortality [948]	mortality [945]
NO2	Short term	mortality [938]	-
	Long term	severe disease [936]	mortality [937,942,946], hospitalization [942], ICU [937,942], MV [937], severe disease [948]
NOx	Long term	ICU [949], mortality [949]	hospitalization [949], ICU [949], mortality [945,949]
O3	Long term	hospitalization [942], ICU [942], mortality [942]	severe disease [936]

Abbreviations: ICU—intensive care unit, MV—mechanical ventilation, BaP—Benzo[a]pyrene, and PM—particulate matter (number indicates upper boundary of particle size in µm).

## Data Availability

Not applicable.

## Supplementary Materials

The following supporting information can be downloaded at: https://www.mdpi.com/article/10.3390/v15010175/s1, Table S1: List of, and compiled results from, selected large cohort studies and meta-analyses investigating multiple risk factors of severe COVID-19.

## Appendix A. Relative Risk Measures Used to Identify COVID-19 Risk Factors

The risk ratio (RR), the odds ratio (OR) and the hazard ratio (HR) are three frequently used statistical measures in medical research that compare the occurrence of a clinical outcome between two groups. These two groups may consist of a control group, and one in which some treatment was administered, or in which a clinically relevant characteristic can be observed (the “intervention group”). However, the exact goal and therefore the use and limitations differ between these statistics.

Risk is the probability of a measured outcome occurring in one of the groups. Hence, the risk ratio (RR) is the quotient of the risks in the intervention and in the control group. A RR = 1 means that there is no difference in the likelihood of the outcome occurring (“null hypothesis”), while a RR > 1 or RR < 1 shows increased or decreased risk in the intervention group compared to the control (the “alternative hypothesis”). The reliability and uncertainty of the result is typically indicated by the *p*-value and the confidence interval. The RR is informative and easy to understand, but its calculation requires a representative sample of the population, and it is therefore not applicable in some study designs (e.g., case–control studies).

Odds are the quotient of the number of events and non-events in one of the studied groups, where the event is the outcome of interest. The odds thus express how many times it is more likely that the outcome happens than it does not, and the odds ratio (OR) measures how much larger the odds are in one of the groups compared to the other. This measure can also be calculated in case–control studies, where a representative sample of the studies population is not available, and the overall risk and odds of the event in either group cannot be directly estimated. On the other hand, the OR is harder to intuitively understand. Importantly, the OR approximates the RR accurately when risks are very small; however, with larger risks, the OR overestimates the RR.

Finally, hazard estimates the instantaneous probability of the observed event during the observation period, as opposed to risk and odds that refer to the occurrence of an event during the entire observation period. The hazard ratio (HR) is therefore the ratio of the hazards in the intervention group and in the control group, and it shows how the intervention changes the rate of experiencing the outcome. HRs are meaningful if the rates are relatively constant in the two groups over the observation period.

For a more detailed explanation and helpful examples, we recommend reading the review by George et al. [1118].
